# Enhancing glymphatic transport through angiotensin II type 2 receptor activation promotes neurological recovery after traumatic brain injury

**DOI:** 10.7150/thno.117743

**Published:** 2025-09-03

**Authors:** Xiaoyu Zhang, Bin Sun, Wenzhong Li, Tianyi Liu, Wenchen Li, Bo Chen, Chuan He, Qin Liu, Shoujun Zhu, Haifeng Wang

**Affiliations:** 1 Department of Neurosurgery, The Second Hospital of Jilin University, Changchun, 130041, P. R. China.; 2 Joint Laboratory of Opto-Functional Theranostics in Medicine and Chemistry, The First Hospital of Jilin University, Changchun, 130021, P. R. China.; 3 Department of Neurosurgery, The First Hospital of Jilin University, Changchun, 130021, P. R. China.

**Keywords:** Traumatic brain injury, Glymphatic transport, AT2R, NIR-II, AQP4

## Abstract

**Background:** Traumatic brain injury (TBI) may impair the function of the glymphatic system, leading to diminished metabolic waste clearance and aggravated neurological deficits. While angiotensin II type 2 receptor (AT2R) activation has demonstrated neuroprotective effects, its specific impact on the glymphatic system following TBI remains uncharacterized.

**Methods:** We utilized near-infrared II (NIR-II) probes with distinct protein-binding capacities to visualize glymphatic transport in TBI mice and investigate how compound 21 (C21)-mediated AT2R activation modulates post-traumatic glymphatic function. Perivascular aquaporin-4 (AQP4) polarization was analyzed by immunofluorescence. RNA sequencing was performed to explore the C21-induced dynamic immune modulation. β-amyloid clearance efficiency and phosphorylated tau accumulation were quantified in mouse brain tissue. Motor and cognitive functions were comprehensively evaluated through standardized behavioral tests.

**Results:** Our results demonstrate that C21-mediated AT2R activation enhanced glymphatic influx and promoted glymphatic clearance after TBI. Mechanistically, AT2R activation restored perivascular aquaporin-4 (AQP4) polarization and cerebral blood flow, suppressed astrogliosis and microglial activation, and attenuated neuroinflammatory responses. Furthermore, AT2R activation enhanced β-amyloid clearance efficiency and reduced phosphorylated tau accumulation, thereby promoting motor and cognitive functional recovery.

**Conclusion:** By employing non-invasive or minimally invasive NIR-II imaging, our study highlights the protective effects of AT2R activation on the glymphatic system following TBI, revealing its potential as a promising therapeutic strategy for mitigating TBI-induced damage and improving neurological outcomes.

## Introduction

Traumatic brain injury (TBI) is a leading neurological disorder and the primary cause of disability and death in individuals under the age of 45 worldwide 1, 2, characterized by acute neurological dysfunction and long-term neurodegenerative pathologies, including cognitive decline, motor deficits, and heightened risks of neurodegenerative diseases 3-5. A critical yet underexplored contributor to these outcomes is the dysregulation of the glymphatic system, a brain-wide perivascular network responsible for cerebrospinal fluid (CSF)-interstitial fluid (ISF) exchange and metabolic waste clearance 6-8. Glymphatic dysfunction after TBI disrupts the removal of neurotoxic proteins such as β-amyloid and phosphorylated tau, exacerbating secondary injury cascades 9-11. Despite its pathophysiological significance, therapeutic strategies targeting glymphatic restoration remain limited.

The glymphatic system, a brain-wide clearance network, operates through astrocyte-defined perivascular channels along penetrating arteries. Its function hinges on two phases: (1) the glymphatic influx phase, during which CSF moves from the subarachnoid space into the brain parenchyma via periarterial spaces penetrating the cortical surface, and (2) the glymphatic clearance phase, during which CSF-ISF mixtures carrying neurotoxic waste (e.g., β-amyloid, phosphorylated tau) exit via perivenous pathways 12-16. Central to this process is the polarized expression of aquaporin-4 (AQP4) water channels at astrocytic end-feet surrounding perivascular spaces (PVS). This spatial polarization enables rapid CSF influx into the parenchyma while directing CSF-ISF efflux toward perivenous spaces, ensuring efficient waste clearance 17-19. Following TBI, glial fibrillary acidic protein (GFAP)-positive reactive astrocytes exhibit aberrant proliferation and activation in the ipsilateral cerebral hemisphere, forming dense glial scars. These pathological alterations disrupt the polarized distribution of AQP4, leading to disorganized localization at perivascular end-feet 11, 20, 21. Such pathological changes impair the glymphatic fluid transport, creating a vicious cycle: impaired waste clearance promotes neuroinflammation, which further damages glymphatic infrastructure. This dysfunction facilitates pathological accumulation of β-amyloid and phosphorylated tau, and ultimately results in chronic neurodegeneration and cognitive decline 10, 11, 21-23. Consequently, enhancing glymphatic transport has emerged as a promising therapeutic target for mitigating TBI-induced neurological damage.

Given the pivotal role of the glymphatic system in TBI pathology, the exploration of potential therapeutic strategies is necessary. Emerging evidence suggests that the brain renin-angiotensin system (RAS), traditionally recognized for cardiovascular regulation, plays a pivotal role in neurological pathologies 24-26. Among its components, the angiotensin II type 1 receptor (AT1R) and angiotensin II type 2 receptor (AT2R), both G protein-coupled receptors, are widely expressed in neurons, astrocytes, oligodendrocytes, and microglia within brain regions such as the cortex, hippocampus, and basal ganglia. During TBI, excessive AT1R activation induces detrimental effects such as reduced cerebral blood flow (CBF), elevated intracranial pressure, cerebral edema, exacerbated neuroinflammation, and long-term cognitive impairment 27-29. Conversely, AT2R activation exerts neuroprotective effects in neurological diseases, promoting neurogenesis, attenuating pathological microglial/astrocytic activation, suppressing pro-inflammatory cytokine release (e.g., IL-1β and TNF-α), and reducing β-amyloid/phosphorylated tau accumulation 30-35. Notably, compound 21 (C21), a highly selective nonpeptide AT2R agonist with 25,000-fold specificity for AT2R over AT1R 36, has demonstrated multifaceted neuroprotective effects in preclinical models of neurodegenerative, neuroinflammatory, and ischemic conditions. We therefore hypothesized that C21-mediated AT2R activation enhances glymphatic transport to alleviate TBI-induced neurological dysfunction.

Technological limitations have hindered real-time assessment of glymphatic system dynamics. Conventional imaging modalities lack the spatiotemporal resolution to capture rapid fluid transport or parenchymal clearance. Recent advances in near-infrared II (NIR-II) fluorescence imaging (1,000-1,700 nm) overcome these barriers, offering deep-tissue penetration, high resolution, and minimal autofluorescence^37^-^40^. This study developed NIR-II probes with adjustable albumin affinity to visualize distinct stages of glymphatic transport: BSA@IR-780 (albumin-bound cyanine) traces CSF influx into PVS and superficial cervical lymph nodes (CLNs); IR-808Ac (albumin-escaping) permits real-time visualization of parenchymal solute clearance; and IR-808 (albumin-seeking) quantifies clearance dynamics from the brain into the blood circulation. This advanced NIR-II imaging platform provided critical methodological support for investigating the effects of C21-mediated AT2R activation on post-TBI glymphatic transport. Our findings demonstrate that AT2R activation enhanced glymphatic transport in TBI mice, concurrent with reduced neuroinflammation, accelerated clearance of neurotoxic proteins, and improved neurological recovery.

## Materials and Methods

### Animals

All C57BL/6 male mice, aged 8-10 weeks, were purchased from Jilin Qianhe Model Biotechnology Co., Ltd. Their weights were maintained between 23 and 25 g. The mice were housed under a standard 12 h light/dark cycle in specific pathogen-free conditions, with controlled temperature (23 ± 1.5 °C) and humidity (50 ± 10%). They had unrestricted access to bedding, nesting materials, food, and water. All mouse experiments were conducted in accordance with the relevant guidelines and regulations of Jilin University and approved by the Animal Ethics Committee of the First Hospital of Jilin University (Scheme No.: 20240624-12). Mice were randomly allocated to different experimental groups, and individuals blinded to the group assignments performed experiments and data collection.

### TBI

The closed-head TBI model was established using a modified weight drop device based on previously described methodologies 41, 42. In contrast to other rodent models, this approach avoided the prolonged anesthesia, thereby more accurately replicating typical clinical conditions. Briefly, mice were anesthetized with 4% isoflurane (ISO) for 1 min, followed by maintenance anesthesia with 1% ISO. After exposing the skull and removing the periosteum, the impact point was marked at 2.0 mm posterior and 2.0 mm lateral to the bregma. The mice were then placed on a rubber surface (6 mm thick) with their heads positioned beneath a weight drop device. A 17.4-g striker (2 mm diameter) was placed perpendicular to the skull at the marked impact point. A 60-g weight was dropped from a height of 15 cm to induce TBI, after which the wound was sutured intermittently. In the TBI models used in this study, no macroscopic evidence of skull fractures or intracranial hemorrhage was observed upon gross examination. The sham group underwent anesthesia and surgical preparation but were not injured.

### Drug administration and experimental design

C21 (HY-100113, MedChemExpress), a highly specific AT2R receptor agonist, was dissolved in phosphate buffered saline (PBS). Mice received intraperitoneal injection of C21 (0.03 mg/kg) or PBS 1 h after TBI, followed by two additional doses at 24 and 48 h post-injury. The selected dosage was determined according to previously established optimal efficacy parameters for stroke treatment 43. The non-injury sham group underwent sham injury and received PBS injections (i.p.).

### Behavioral tests

Behavioral assessments included the modified neurological severity score (mNSS), rotarod test, novel object recognition (NOR) test, open field test (OFT) and Morris water maze (MWM) test (methodological details in Supplementary Information).

### Synthesis of probes

BSA@IR-780, IR-808, and IR-808Ac were synthesized following previously methods 40, 44.

### Construction of the NIR-II fluorescence imaging system

For detailed NIR-II imaging setup procedures, refer to relevant classical literature 45, 46. This study utilized a two-dimensional InGaAs array (Raptor Photonics) to collect NIR-II fluorescence signals. All imaging employed an 808 nm laser (power density ~65 mW/cm²) irradiated through an 850 nm short-pass filter. Emission from BSA@IR-780, IR-808, and IR-808Ac was isolated using 1000 nm + 1100 nm long-pass filters, ensuring signal capture within the NIR-II region. A short-wave infrared lens (35 mm focal length) mounted on the detector provided fields of view of ~2.8 × 2.3 cm². The imaged region remained consistently centered within the laser illumination area. Computer-controlled image exposure times are specified in the corresponding figure legends.

### Intracisternal tracer infusions

Under ISO anesthesia, mice were fixed in a stereotactic frame. BSA@IR-780 was dissolved in artificial CSF at a concentration of 300 μM. After surgically exposing the cisterna magna (CM), a PE10 tube filled with BSA@IR-780 was inserted into the CM using a 30-gauge needle bridge. The inserted PE10 tube was first sealed with 3M vetbond tissue adhesive and reinforced with dental cement. Subsequently, dexmedetomidine (DEX) was dissolved in PBS and injected intraperitoneally (0.2 mg/kg). After 5 min, a syringe pump (Harvard Instruments) was used to deliver 7 μL of BSA@IR-780 at a rate of 1 μL/min. A heating pad was used to maintain the body temperature at 36.5-37 °C.

### Visualization of BSA@IR-780 influx into brain

The influx of BSA@IR-780 into the mouse brain was monitored using an NIR-II fluorescence imaging system we had constructed. The brain was focused on the field view of ~2.8 × 2.3 cm². The distribution was monitored for 30 min with an interval of 5 min. After allowing the NIR-II tracer to circulate for 30 min, the brain was collected and the tracer distribution on the dorsal and ventral brain surfaces was imaged. The imaging conditions were 10 ms, 1200 nm long-pass filter, and 65 mW/cm^2^ power density.

The brain was then fixed with 4% paraformaldehyde (PFA) for 24 h. Coronal slices (100-μm thick) were prepared using a vibratome. A total of twelve slices were collected at 300 μm intervals, starting 1.6 mm anterior to bregma, to calculate the mean total tracer influx for the entire brain. The slices were imaged using a scanning imaging system (Azurespot).

### Visualization of the BSA@IR-780 drainage into superficial CLNs

Mouse hair was shaved prior to imaging to expose the neck skin. Infusions were performed with mice in a prone position. After completing intracisternal infusion of BSA@IR-780, mice were placed supine for superficial CLNs imaging. The first image was acquired 10 min post-infusion start, with tracer movement monitored for 60 min at 5-min intervals. Imaging parameters: 10-ms exposure, 1200 nm long-pass filter, 65 mW/cm² power density.

### Intra-parenchymal injections

To evaluate the clearance capacity of parenchymal metabolites, 2 μL of the NIR-II probe IR-808Ac (50 μM, dissolved in artificial CSF) or IR-808 (2.5 μM, dissolved in artificial CSF) was injected into the cerebral cortex of mice using stereotaxic technique. Mice were anesthetized with 4% ISO for 1 min, followed by maintenance with 1% ISO. The skull was exposed, and the periosteum was removed. A 33-gauge stainless-steel cannula (Hamilton) was inserted into the cerebral cortex at the following stereotactic coordinates: 2.00 mm posterior, 1.50 mm lateral to the bregma, and 2.00 mm below the brain surface. After 5 min of intubation, the injection was administered at 0.2 μL/min. After the injection, the needle was removed following a 5-min rest period, and NIR-II imaging of the mouse brain was conducted immediately.

FITC-labeled β-amyloid (1-42) (P2000022-FITC, PLLABS) was dissolved in artificial CSF at a concentration of 0.5 mg/mL. A 2-μL volume of the solution was injected into the brain parenchyma of mice using the same technique. The mice were then returned to their cages and allowed to move freely for 4 h. Subsequently, mice were anesthetized with chloral hydrate and perfused with cold PBS. The brains were harvested and fixed in 4% PFA at 4 °C for 24 h, sectioned into 100-μm thick slices, and the residual β-amyloid on the slices was observed under a confocal microscope (FV3000, Olympus).

### Visualization of NIR-II probes clearance from brain

Using our custom NIR-II fluorescence imaging system, we monitored IR-808Ac clearance from the mouse brain within a field of view of ~2.8 × 2.3 cm². Immediately after injecting IR-808Ac into the parenchyma, we performed the initial NIR-II brain imaging. Mice were then returned to their cages without activity restrictions, with two additional imaging sessions conducted at 1-h intervals. All *in vivo* imaging used identical parameters: 15 ms exposure, 1200 nm long-pass filter, and 65 mW/cm² power density.

The percentage of IR-808Ac cleared from the mouse brain was calculated as clearance efficiency using the following formula:

Clearance efficiency (%) = [(initial fluorescence intensity - current fluorescence intensity) / initial fluorescence intensity] × 100

Then, the brain was collected. Both dorsal and ventral surfaces were imaged (dorsal: 10 ms, 1200 nm long-pass filter, 65 mW/cm²; ventral: 100 ms, 1200 nm long-pass filter, 65 mW/cm²) to assess the residual tracer. Brains were then fixed in 4% PFA for 24 h. Using a vibratome, we prepared 100-μm-thick coronal slices. Six slices, collected at 300-μm intervals starting 0.8 mm posterior to bregma, were imaged using an Azurespot scanning system.

After intraparenchymal injection of IR-808, the tracer was cleared into the peripheral circulation. IR-808 accumulation in the femoral vein was monitored using our NIR-II fluorescence imaging system within a field of view of ~2.8 × 2.3 cm². Prior to imaging, the mouse leg was shaved to expose the skin. Immediately following IR-808 injection, the mouse was positioned supine for imaging of the leg vasculature. Continuous monitoring was performed for 60 min at 5-min intervals under the following imaging parameters: 100 ms exposure, 1200 nm long-pass filter, and 65 mW/cm² power density.

### Immunofluorescence

Mice were deeply anesthetized and perfused with cold PBS. Their brains were removed and fixed in 4% PFA at 4 °C overnight, then dehydrated in 15% and 30% sucrose solutions. After dehydration, the brains were immersed in optimal cutting temperature compound (Sakura Finetek USA, Torrance, California, USA) and sectioned into 30-μm slices using a cryostat microtome (CM1950, Leica Biosystems, Nurock, Germany). The tissue sections were washed with PBS and sealed with PBS containing 3% bovine serum albumin, 0.2% Triton X-100, and 0.05% Tween 20 at room temperature for 1.5 h. Sections were incubated overnight at 4 °C with primary antibodies, followed by a 2-h incubation at room temperature with secondary antibodies. The primary antibodies used were rabbit anti-AQP4 (1:500, CL488-16473, Proteintech), hamster (Armenian) anti-CD31 (1:400, MAB1398Z, Merck), chicken anti-GFAP (1:500, PA1-10004, Thermo Fisher Scientific), and rabbit anti-Iba1 (1:500, AB178846, Abcam). Stained sections were observed under a confocal microscope (FV3000, Olympus).

### AQP4 polarization evaluation

To assess AQP4 polarization after TBI, we employed an established area-based method 23, 47. Briefly, we measured the median AQP4 immunofluorescence intensity within the perivascular region. Using threshold analysis, we determined the percentage of area where AQP4 immunofluorescence intensity met or exceeded the levels observed around blood vessels (termed AQP4% area). Polarization was then calculated as: Polarization = 1 - AQP4% area. This value represents the percentage of area exhibiting AQP4 immunoreactivity lower than that in the perivascular astrocytic end-feet.

As an alternative approach, we quantified AQP4 polarization using immunofluorescence signals for AQP4 and the vascular marker CD31, following previous researches 48-50. Briefly, lines perpendicular to blood vessels were randomly selected. AQP4 fluorescence intensity profiles were recorded along these lines, with signal within the vessel lumen representing the baseline intensity. Polarization was calculated as the ratio of peak AQP4 fluorescence intensity at perivascular end-feet to the average baseline intensity:

AQP4 polarization = perivascular peak intensity / average baseline intensity

### Measurement of CBF

CBF was measured using a laser Doppler blood flow meter. Briefly, after anesthetizing the mice as described earlier, a midline scalp incision was made to expose the skull. The CBF of both hemispheres was then recorded using a laser speckle imager.

### Measurement of mean arterial pressure (MAP)

MedLab non-invasive blood pressure analysis system (NJKEWBIO, Nanjing, China) was used to measure the MAP of mice. Place the mouse on a 37-39°C heating platform or in a thermostatic chamber for 5-10 min to promote tail vasodilation and enhance signal clarity. Gently restrain the mouse in a specialized holder, ensuring natural tail extension through the tail cuff sensor. Maintain unobstructed respiration by avoiding thoracic compression. Clean the tail with alcohol swabs to remove debris, then position the tail cuff approximately 1-2 cm from the tail base. Adjust cuff tension to achieve effective blood flow occlusion without tissue compression. Initialize the system and perform baseline calibration as per software instructions. The automated protocol initiates with cuff inflation to 150 mmHg (surpassing typical systolic pressure). Gradual deflation (2-3 mmHg/s) enables detection of hemodynamic parameters: systolic blood pressure (SBP) corresponds to the pulse wave reappearance point, while diastolic blood pressure (DBP) is identified at complete blood flow restoration. Conduct triplicate measurements, discarding outliers before calculating mean values. Derive MAP using the standard formula:

MAP = DBP + 1/3(SBP - DBP)

### Heart rate and respiratory rate monitoring

The methods for measuring heart rate and respiratory rate in mice were consistent with those described in previous studies 40. It is important to note that the mice used for physiological signal measurements and those used for imaging were separate cohorts within the same experimental group. For heart rate measurement, electrodes were attached to the limbs of the mice, and ECG signals were collected using a non-invasive small animal multifunctional physiological monitoring system (SftReplay). The average value over a 2-min recording period was used to calculate the heart rate of each mouse. For respiratory rate measurement, each mouse was placed in a prone position, and a respiratory sensor was positioned on the abdomen. Respiratory data were continuously collected for 2 min, and the average value was used to determine the respiratory rate of each mouse. To ensure accurate readings, the body temperature of the mice was maintained at 36.5-37.0°C using a thermostatically controlled heating pad, thereby minimizing potential confounding effects on physiological measurements.

### RNA sequencing (RNA-seq)

Total RNA was extracted from the cortical tissue using TRIzol® Reagent according to the manufacturer's instructions (Magen). RNA samples were detected based on the A260/A280 absorbance ratio with a Nanodrop ND-2000 system (Thermo Scientific, USA), and the RIN of RNA was determined by an Agilent Bioanalyzer 4150 system (Agilent Technologies, CA, USA). Only qualified samples will be used for library construction. Paired-end libraries were prepared using ABclonal mRNA-seq Lib Prep Kit (ABclonal, China) following the manufacturer's instructions. The mRNA was purified from 1 μg total RNA using oligo (dT) magnetic beads followed by fragmentation carried out using divalent cations at elevated temperatures in ABclonal First Strand Synthesis Reaction Buffer. Subsequently, first-strand cDNAs were synthesized with random hexamer primers and Reverse Transcriptase (RNase H) using mRNA fragments as templates, followed by second-strand cDNA synthesis using DNA polymerase I, RNAseH, buffer, and dNTPs. The synthesized double stranded cDNA fragments were then adapter-ligated for preparation of the paired-end library. Adaptor-ligated cDNA was used for PCR amplification. PCR products were purified (AMPure XP system) and library quality was assessed on an Agilent Bioanalyzer 4150 system. Finally, sequencing was performed with an Illumina Novaseq 6000/MGISEQ-T7 instrument. The data generated from Illumina/BGI platform were used for bioinformatics analysis.

### Western blot

Mice were perfused with cold PBS, and their brains were extracted. Tissue samples were collected from near the cerebral cortex around the injury site. Total proteins were extracted from each group using protease and phosphatase inhibitors (P1260, Beijing, China) and loaded onto 10% or 12% SDS-PAGE gels. Proteins were then transferred on polyvinylidene fluoride (PVDF) membranes using a wet electrotransfer device (Bio-Rad). The PVDF membranes were incubated overnight at 4 °C with specific primary antibodies, including: rabbit anti-pERK1/2 (Thr202/Tyr204) (1:3000, 28733-1-AP, Proteintech), rabbit anti-ERK1/2 (1:3000, A4782, ABclonal), mouse anti-Tau5 (1:2000, MA5-12808, Thermo Fisher Scientific), rabbit anti-pTau-Thr205 (1:2000, AP0168, ABclonal), rabbit anti-pTau-Ser396 (1:2000, 9632T, Cell Signaling Technology), and rabbit anti-pTau-Ser404 (1:2000, 44-758G, Thermo Fisher Scientific). After incubation, the membranes were washed thrice. After washing, membranes were incubated with horseradish peroxidase-conjugated secondary antibodies, and protein bands were detected using an enhanced chemiluminescence system (MilliporeSigma).

### Statistical analysis

All image analyses were performed using ImageJ, and statistical analyses were conducted with GraphPad Prism 8. Data in all graphs are presented as mean ± standard deviation (SD), with individual data points and lines representing values from each mouse. Comparisons between two groups were performed using Student's t-test, whereas one-way ANOVA followed by Tukey's post-hoc test was applied for comparisons across three or more groups. Two-way ANOVA was employed to analyze interactions between continuous variables. For datasets violating assumptions of normality or homogeneity of variances, the nonparametric Kruskal-Wallis test was used to compare medians across three groups, followed by Dunn's test for multiple comparisons. Statistical significance was defined as *p* < 0.05.

## Results

### AT2R activation improved neurological recovery after TBI

**Figure 1:**
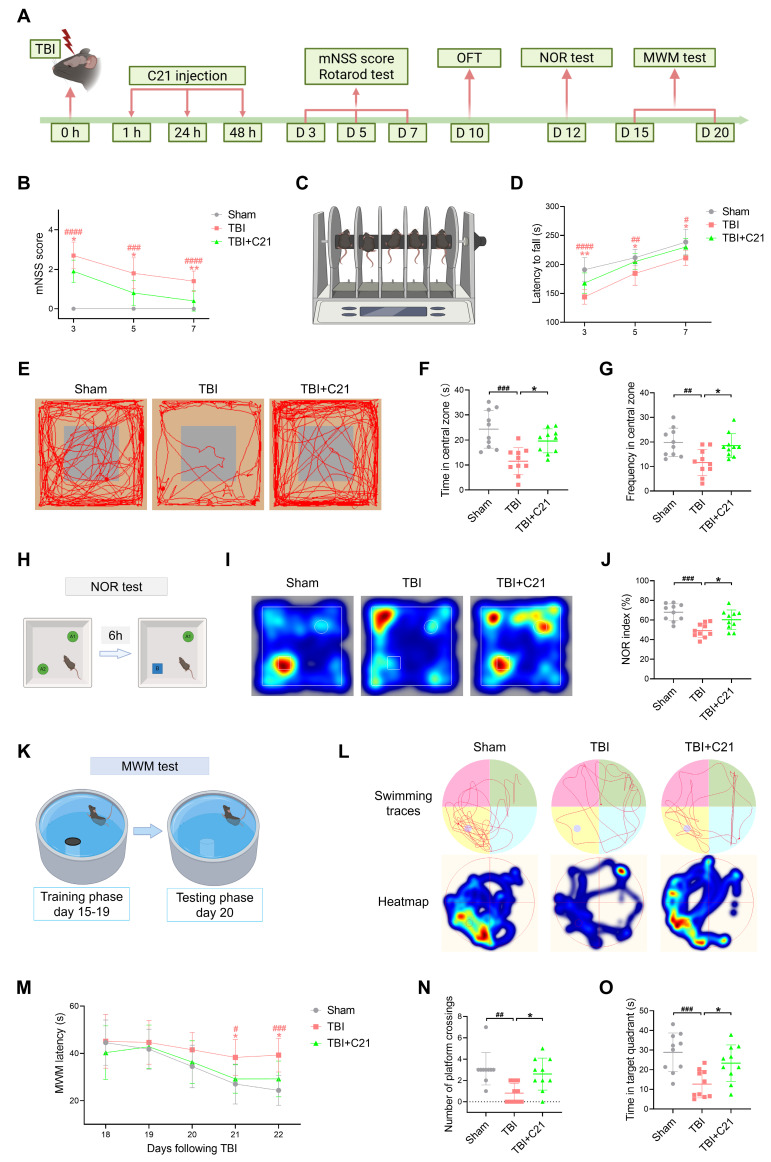
** AT2R activation improved neurological recovery after TBI. (A)** Workflow detailing the timeline for drug interventions and behavioral tests. **(B)** mNSS after TBI.** (C, D)** Latency to fall on the rotarod test after TBI. **(E)** Representative track sheets from the open-field test probe trial.** (F, G)** OFT results: Time spent in the central zone **(F)** and frequency of entries into the central zone **(G)**. **(H)** Schematic diagram representation of the NOR test. The schematic diagram was created with BioRender.com. A1 and A2 represent old objects, while B represents the novel object. **(I)** Representative heatmaps from the NOR test probe trial. **(J)** NOR index for each group. **(K)** Schematic diagram of the MWM test. The schematic diagram was created with BioRender.com. **(L)** Representative swimming traces and heatmaps from the MWM test. **(M)** Learning curve during the training phase of the MWM test. **(N, O)** MWM probe trial results: number of platform crossings **(N)** and time spent in the target quadrant **(O)** for each group. All data are presented as mean ± SD (n = 10 per group). Statistical significance: #p < 0.05, ##p < 0.01, ###p < 0.001, ####p < 0.0001 versus sham group; *p < 0.05, **p < 0.01, ***p < 0.001, ****p < 0.0001 versus TBI+C21 group.

To investigate the impact of AT2R activation on neurological recovery after TBI, we performed behavioral tests assessing motor and cognitive functions (Figure 1A). Results from the mNSS and rotarod test revealed significant motor deficits post-TBI, while C21-treated mice exhibited progressive improvement in motor performance on days 3, 5, and 7 post-TBI (Figure 1B-D). For cognitive assessment, the OFT, NOR test, and MWM test were employed. The OFT was used to evaluate exploratory behavior in mice. Compared with the sham group, the TBI group spent less time in the central area and exhibited fewer crossings, indicating reduced exploratory activity. Treatment with C21 increased both the time spent in and crossings through the central area in TBI mice (Figure 1E-G). The NOR test was used to assess memory and object recognition. TBI mice spent less time near the new object compared with the sham group. Treatment with C21 increased the NOR index (Figure 1H-J). The MWM test assessed spatial learning and memory. During training sessions on days 18 and 19 post-injury, TBI+C21 mice showed shorter escape latencies than untreated TBI mice. (Figure 1K-M). On day 20, following platform removal, the TBI+C21 group crossed the former platform location more frequently and spent more time in the target quadrant (Figure 1N, O), suggesting enhanced spatial memory.

### AT2R activation enhanced glymphatic influx in acute TBI mice

**Figure 2:**
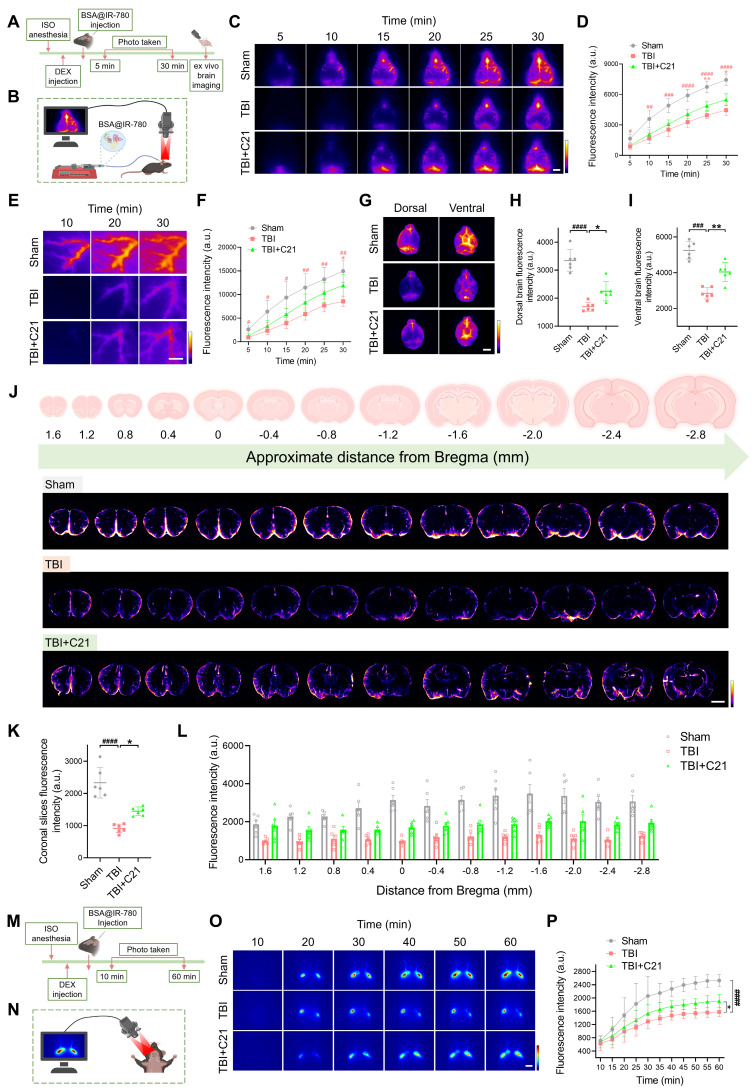
** AT2R activation enhanced glymphatic influx in acute TBI mice. (A, B)** Experimental workflow (A) and schematic diagram (B) of transcranial NIR-II imaging to assess glymphatic influx. The schematic diagram was created with BioRender.com. **(C, D)** High-contrast transcranial imaging (C) and quantification of fluorescence intensity (D) for BSA@IR-780. Scale bar = 4 mm. **(E, F)** Representative images of BSA@IR-780 influx into the PVS (E) and corresponding fluorescence quantification (F). Scale bar = 2 mm. **(G)** Representative images of BSA@IR-780 distribution on dorsal and ventral brain surfaces. Scale bar = 4 mm.** (H, I)** Quantified fluorescence intensity on the dorsal (H) and ventral (I) surfaces. **(J)** Representative images of coronal slices at the indicated distances from bregma. Scale bar = 2 mm. **(K, L)** Quantified fluorescence intensity across all slices (K) and per slice (L). **(M, N)** Workflow (M) and schematic diagram (N) for non-invasive NIR-II imaging of superficial CLNs drainage in mice. The schematic diagram was created with BioRender.com. **(O, P)** Representative images (O) and fluorescence quantification (P) of superficial CLNs drainage. Scale bar = 4 mm. All data are presented as mean ± SD (n = 6 per group). Statistical significance: #p < 0.05, ##p < 0.01, ###p < 0.001, ####p < 0.0001 versus sham group; *p < 0.05, **p < 0.01, ***p < 0.001, ****p < 0.0001 versus TBI+C21 group.

The restoration of glymphatic transport following TBI plays a critical role in neurological recovery 10. To evaluate glymphatic influx in mice 3 days after TBI, 7 μL of BSA@IR-780 tracer (see Figure S1 for mass spectrometry) was injected into the CM (Figure 2A, B). Using NIR-II imaging, we dynamically tracked parenchymal penetration of BSA@IR-780 over 30 min. Consistent with TGN-020's known inhibition of AQP4-dependent glymphatic transport 19, 51, TGN-020 suppressed BSA@IR-780 influx (Figure S2). Compared to sham controls, TBI mice exhibited reduced BSA@IR-780 influx into PVS on the brain surface and diminished parenchymal diffusion, indicating TBI-induced glymphatic impairment. C21 treatment restored the influx of BSA@IR-780 into PVS on the brain surface and enhanced its diffusion into the brain parenchyma (Figure 2C-F).

Following 30 minutes of *in vivo* NIR-II imaging, the brains were harvested and imaged *ex vivo* using the same modality. Imaging of the whole brains confirmed the *in vivo* findings. The fluorescence intensity of the BSA@IR-780 probe on both the dorsal and ventral sides of the brain of TBI mice was weaker than that of sham mice. However, treatment with C21 enhanced fluorescence intensity on both the dorsal and ventral sides of the brain, reflecting improved glymphatic influx (Figure 2G-I). To further verify these results, coronal slices were prepared from specific brain regions, and the distribution of BSA@IR-780 within the slices was analyzed (Figure 2J). The results revealed that C21 treatment increased the distribution of BSA@IR-780 in the brain parenchyma (Figure 2K, L).

After injection of BSA@IR-780, we used NIR-II imaging to quantitatively assess its drainage into the superficial CLNs (Figure 2M, N). The results showed that TBI mice exhibited reduced tracer drainage compared to sham-operated mice. However, C21 administration enhanced this drainage (Figure 2O, P).

### AT2R activation promoted glymphatic clearance in acute TBI mice

**Figure 3:**
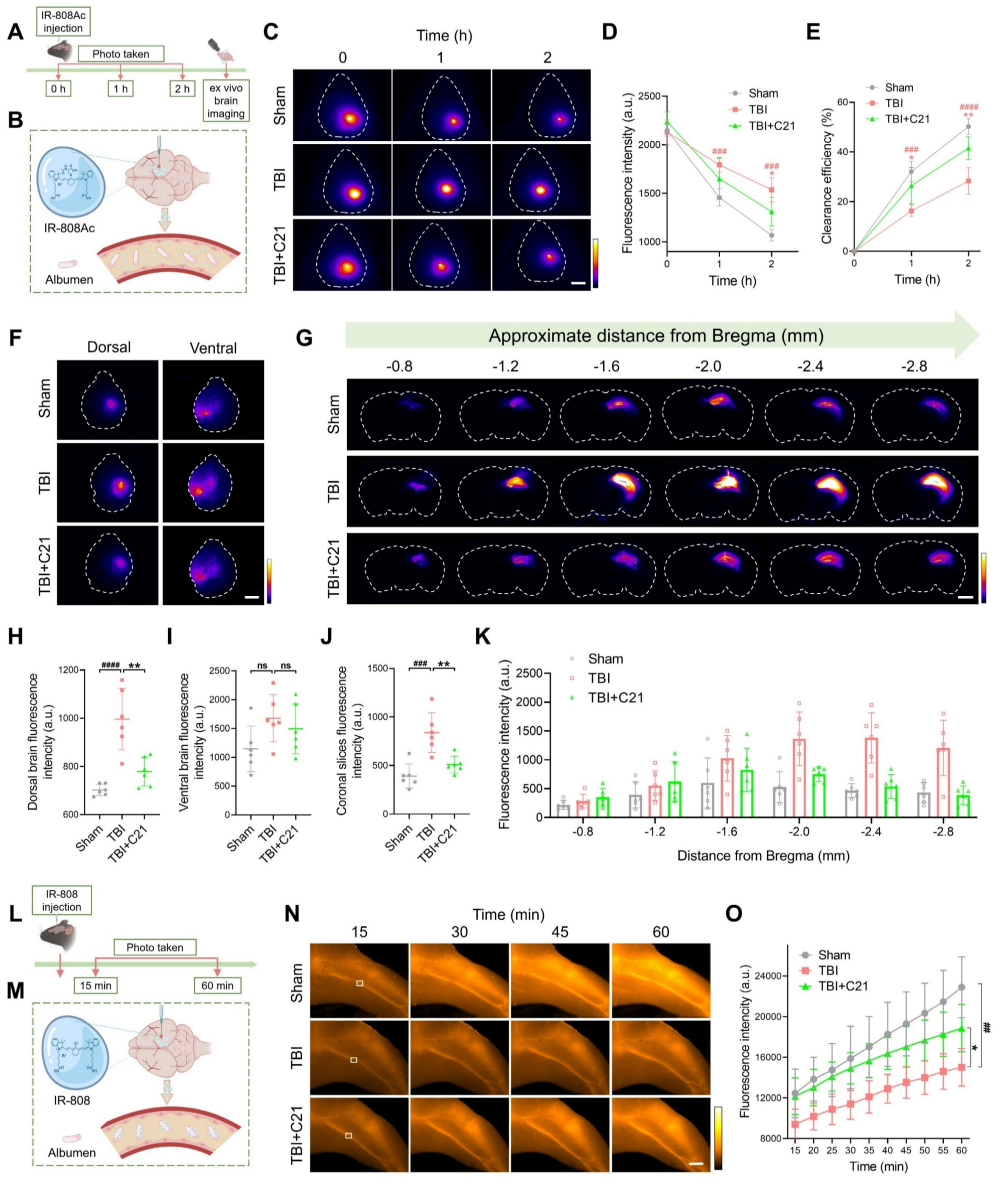
** AT2R activation promoted glymphatic clearance in acute TBI mice. (A, B)** Experimental workflow (A) and schematic diagram (B) for transcranial NIR-II imaging of parenchymal IR-808Ac clearance. The schematic diagram was created with BioRender.com. **(C-E)** High-contrast transcranial NIR-II imaging (C) and fluorescence intensity analysis of IR-808Ac (D, E). Scale bar = 4 mm. **(F)** Representative images of IR-808Ac distribution on dorsal and ventral brain surfaces. Scale bar = 4 mm. **(G)** Representative images of coronal slices at the indicated distances from bregma. Scale bar = 2 mm. **(H, I)** Quantified fluorescence intensity on the dorsal (H) and ventral (I) surfaces. **(J, K)** Quantified fluorescence intensity across all slices (J) and per slice (K). **(L, M)** Workflow (L) and schematic diagram (M) illustrating IR-808 delivery and detection in the femoral vein. The schematic diagram was created with BioRender.com. **(N, O)** Representative femoral vein images (N) and fluorescence quantification (O) of IR-808. Scale bar = 4 mm. All data are presented as mean ± SD (n = 6 per group). Statistical significance: #p < 0.05, ##p < 0.01, ###p < 0.001, ####p < 0.0001 versus sham group; *p < 0.05, **p < 0.01, ***p < 0.001, ****p < 0.0001 versus TBI+C21 group.

To assess the effect of AT2R activation on glymphatic clearance in mice 3 days post-TBI, we first evaluated the parenchymal clearance of IR-808Ac (Figure 3A; ¹H NMR spectrum shown in Figure S3). This NIR-II probe enables transcranial imaging and is rapidly metabolized due to its albumin-escaping capacity (Figure 3B) 44. After cortical injection of IR-808Ac, the parenchymal clearance efficiency was monitored by NIR-II imaging. Consistent with TGN-020's known disruption of glymphatic function via AQP4 inhibition 19, 51, TGN-020 suppressed parenchymal clearance of IR-808Ac (Figure S4). Compared with the sham group, TBI impaired IR-808Ac clearance, whereas C21 treatment improved parenchymal clearance efficiency (Figure 3C-E).

These findings were further validated using *ex vivo* brain imaging (Figure 3F). In TBI mice, the residual IR-808Ac in the dorsal side of the brain was higher than that in sham mice. However, treatment with C21 reduced the residual tracer (Figure 3H). Although C21 treatment also slightly reduced the residual tracer on the ventral side of the brain, the difference compared to TBI mice was not statistically significant (Figure 3I). Coronal slices imaging (Figure 3G) supported these observations, showing that C21 treatment effectively decreased residual IR-808Ac in the brain parenchyma compared to TBI mice (Figure 3J, K).

We further utilized IR-808 to assess glymphatic clearance (Figure 3L). Unlike IR-808Ac, IR-808 has strong albumin-seeking capacity, enabling it to bind to blood albumin and persist in systemic circulation for an extended period (Figure 3M) 44, 52. This property makes IR-808 a suitable tracer for specifically monitoring its clearance from the brain parenchyma into the blood circulation. In this study, after IR-808 was injected into the cerebral cortex, NIR-II *in vivo* imaging was used to continuously monitor fluorescence intensity in the femoral vein. Images were captured from the same location in the femoral vein (Figure 3N), and fluorescence changes were analyzed. The results showed that the fluorescence intensity of IR-808 in the femoral vein of TBI mice was lower than that in sham-operated mice, suggesting impaired glymphatic clearance. However, treatment with C21 notably increased the fluorescence intensity of IR-808 in the femoral veins of TBI mice (Figure 3O), demonstrating improved glymphatic clearance.

### AT2R activation restored AQP4 polarization and CBF in acute TBI mice

**Figure 4:**
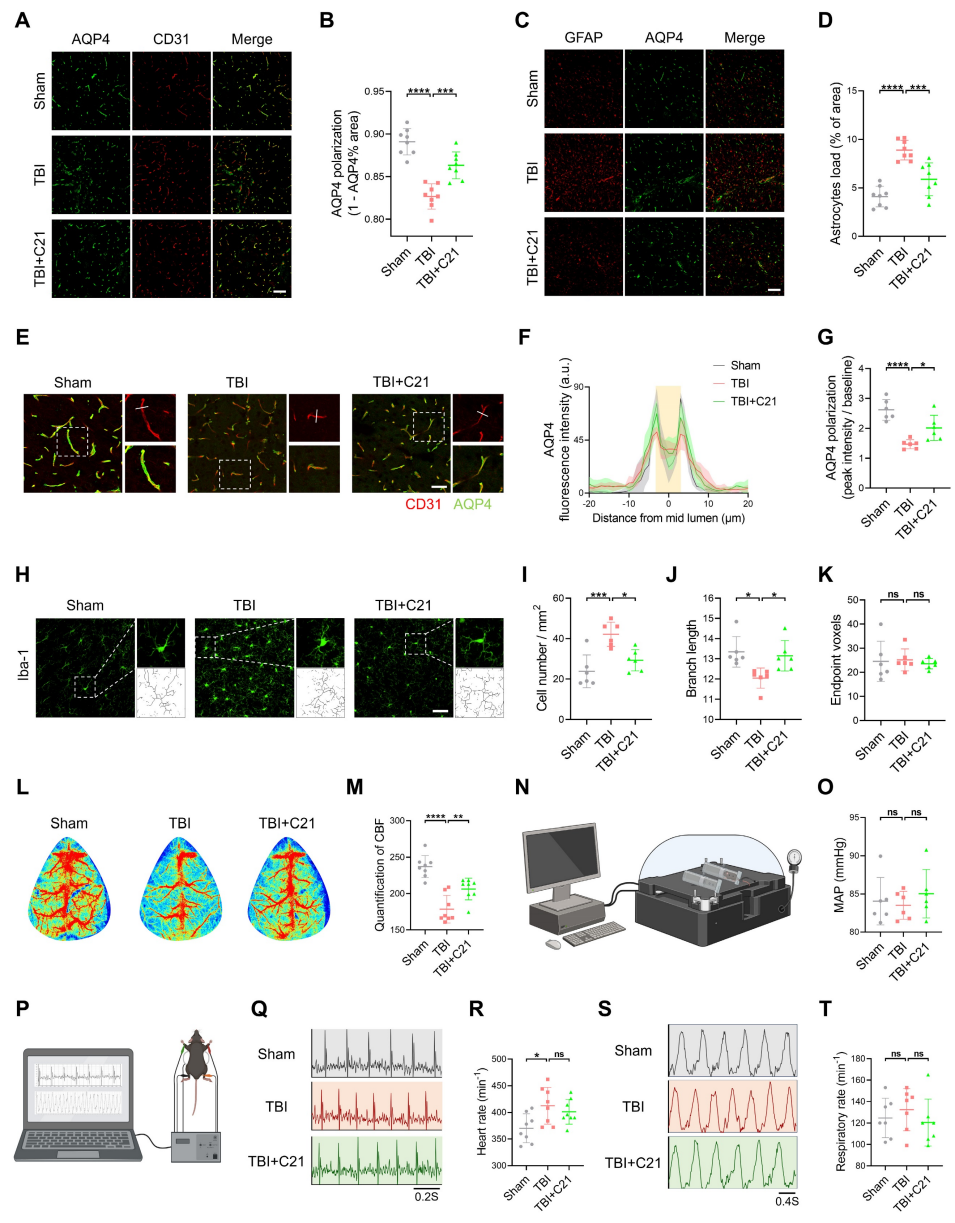
** AT2R activation restored AQP4 polarization and CBF in acute TBI mice. (A)** Co-immunofluorescence staining of AQP4 (green) and CD31 (red) in the perilesional cortex. Scale bar = 100 μm. **(B)** Quantification of AQP4 polarization (AQP4 polarization = 1 - AQP4% area). **(C)** Co-immunofluorescence staining of AQP4 (green) and GFAP (red) in the perilesional cortex. Scale bar = 100 μm. **(D)** Quantification of the GFAP coverage area fraction. **(E)** Representative images of AQP4 (green) and CD31 (red) immunofluorescence staining. Scale bar = 50 μm. **(F)** Quantification of AQP4 fluorescence intensity profile along the white line in (E). **(G)** Quantification of AQP4 polarization (AQP4 polarization = vessel AQP4 peak intensity / average baseline intensity). **(H)** Representative Iba-1 immunofluorescence images and cytoskeletal morphology analysis in the perilesional cortex. Scale bar = 50 μm. **(I-K)** Quantitative analysis of microglial density (I), branch length (J), and endpoint voxels (K). **(L, M)** Representative images and quantification of CBF for all groups. **(N, O)** Measurements and quantification of MAP for all groups. **(P)** Schematic diagram of measuring heart rate and respiratory rate in mice. The schematic diagram was created with BioRender.com. **(Q, R)** Measurements of heart rate for all groups. **(S, T)** Measurements of respiratory rate for all groups. All data are presented as mean ± SD (n = 6-8 per group). Statistical significance: *p < 0.05, **p < 0.01, ***p < 0.001, ****p < 0.0001.

Decreased AQP4 polarization is a key mechanism underlying glymphatic system dysfunction after TBI 11. To investigate the mechanism of AT2R activation on glymphatic transport in acute TBI mice, we analyzed AQP4 polarization in the perilesional cortex 3 days after TBI using double immunofluorescence staining for CD31 (a vascular endothelial marker) and AQP4. The results revealed that C21 treatment effectively reversed the TBI-induced decrease in AQP4 polarization (Figure 4A, B, E-G). Given that extensive reactive astrogliosis following TBI is closely associated with the loss of perivascular AQP4 polarization, we examined the expression of GFAP and AQP4 by immunofluorescence imaging. The results demonstrated increased GFAP expression in the TBI group compared to the sham-operated group. C21 treatment reduced GFAP expression, suggesting attenuation of reactive astrogliosis (Figure 4C, D).

Under pathological conditions, the glymphatic system modulates microglial activation in the perilesional cortex 53. Microglial activation was assessed by measuring the number of endpoint voxels and the average branch length of microglial cells, as activated microglia typically exhibit shortened branches and reduced endpoint voxels 54. Using immunofluorescence staining for Iba1, we analyzed the number and morphology of microglia in brain tissue (Figure 4H; see supplementary methods for quantification of microglial activation). The results showed a significant increase in microglial density and a marked reduction in branch length in the TBI group compared with the sham group, reflecting heightened microglial activation. Treatment with C21 decreased microglial density and restored branch length, suggesting suppressed microglial activation (Figure 4I, J). However, there was no significant difference in endpoint voxel counts among these groups (Figure 4K).

The regulation of the glymphatic system involves dynamic interactions between blood pressure and CBF 55-58. Additionally, emerging evidence suggests that cardiopulmonary rhythms (heart rate/respiratory rate) may modulate glymphatic drainage 59. Therefore, we assessed cardiovascular parameter responses to TBI and C21 intervention. Our findings demonstrated that TBI significantly reduced CBF, whereas C21 treatment effectively restored post-TBI CBF levels (Figure 4L, M). Notably, C21 administration did not induce detectable alterations in MAP, heart rate, or respiratory rate in TBI mice (Figure 4N-T). Furthermore, C21 injection in healthy control mice elicited no significant changes in heart rate, respiratory rate, MAP, or CBF (Figure S5).

### AT2R activation inhibited neuroinflammatory responses in acute TBI mice

**Figure 5:**
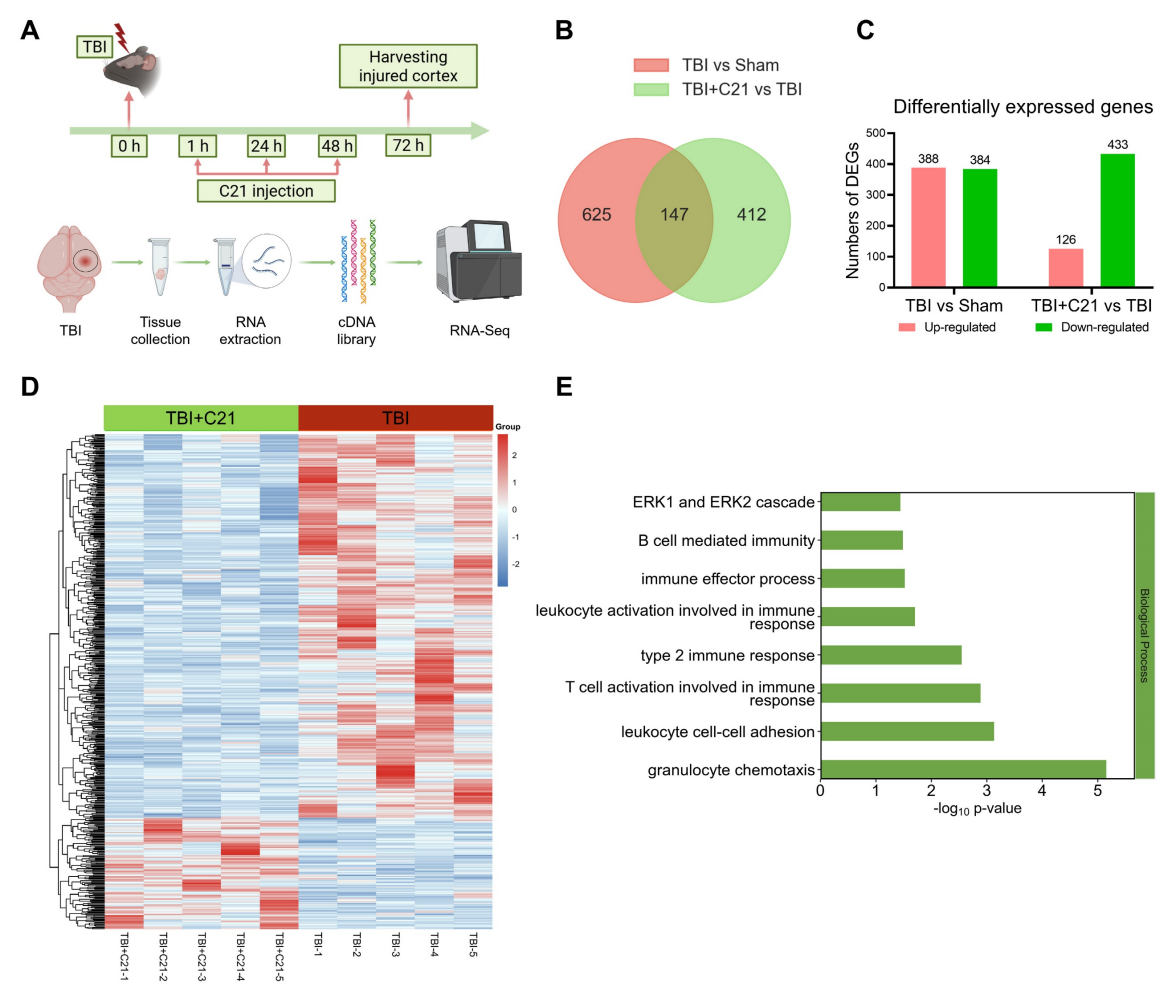
** AT2R activation inhibited neuroinflammatory responses in acute TBI mice. (A)** Flowchart of the RNA-seq analysis. **(B)** Venn diagram illustrating genetic variations. **(C)** Statistically up-regulated and down-regulated DEGs. **(D)** Cluster map of the DEGs among TBI+C21 group and TBI group. **(E)** Down-regulated biological process of gene ontology function enrichment of the DEGs in the TBI+C21 group compared with the TBI group. n = 5 per group.

RNA-seq analysis of the perilesional cortex at 3 days post-TBI revealed a dynamic immune modulation induced by C21 treatment (Figure 5A). High inter-replicate correlation confirmed experimental reproducibility (Figure S6), while principal component analysis demonstrated clear disparities in gene expression profiles among Sham-operated, TBI, and TBI+C21 groups (Figure S7). Comparative analysis identified 772 differentially expressed genes (DEGs) between TBI and Sham-operated groups (388 upregulated, 384 downregulated; Figure 5B, C). The cluster map displayed the DEGs between the TBI and Sham-operated groups (Figure S8A). Gene ontology analysis of TBI-associated DEGs revealed marked enrichment in immune activation pathways, including B cell-mediated immunity, T cell activation involved in immune response, leukocyte activation involved in immune response, leukocyte cell-cell adhesion, granulocyte chemotaxis, and ERK1/2 cascade (Figure S8B). Notably, C21 administration substantially attenuated TBI-induced transcriptional alterations, with 559 DEGs distinguishing TBI+C21 from TBI groups (126 upregulated, 433 downregulated; Figure 5B, C). The cluster map displayed the DEGs between the TBI+C21 and TBI groups (Figure 5D). Crucially, C21 treatment reversed TBI-associated immune pathways, including B cell-mediated immunity, T cell activation involved in immune response, leukocyte activation involved in immune response, leukocyte cell-cell adhesion, granulocyte chemotaxis, and ERK1/2 cascade (Figure 5E). Together, these findings demonstrate that C21 treatment ameliorated neuroinflammatory responses initiated by TBI.

In addition, RNA-seq analysis revealed that the AT2R signaling pathway involves the ERK1/2, a key regulator of brain injury and repair 60. To investigate the potential signaling pathways mediating the protective effects of AT2R, we examined the effect of C21 on ERK1/2 activation in the injured cortex in mice 3 days after TBI using Western blot. Our results revealed that C21 led to a significant decrease in ERK1/2 (Thr202/Tyr204) phosphorylation (Figure S9), suggesting reduced activation of ERK1/2. Subsequently, we investigated whether inhibiting ERK activation would affect CBF and AQP4 polarization. The results revealed that administration of the ERK inhibitor U0126 promoted the restoration of both AQP4 polarization and CBF in mice 3 days post-TBI (Figure S10).

### AT2R activation suppressed β-amyloid and phosphorylated tau accumulation in chronic TBI mice

**Figure 6:**
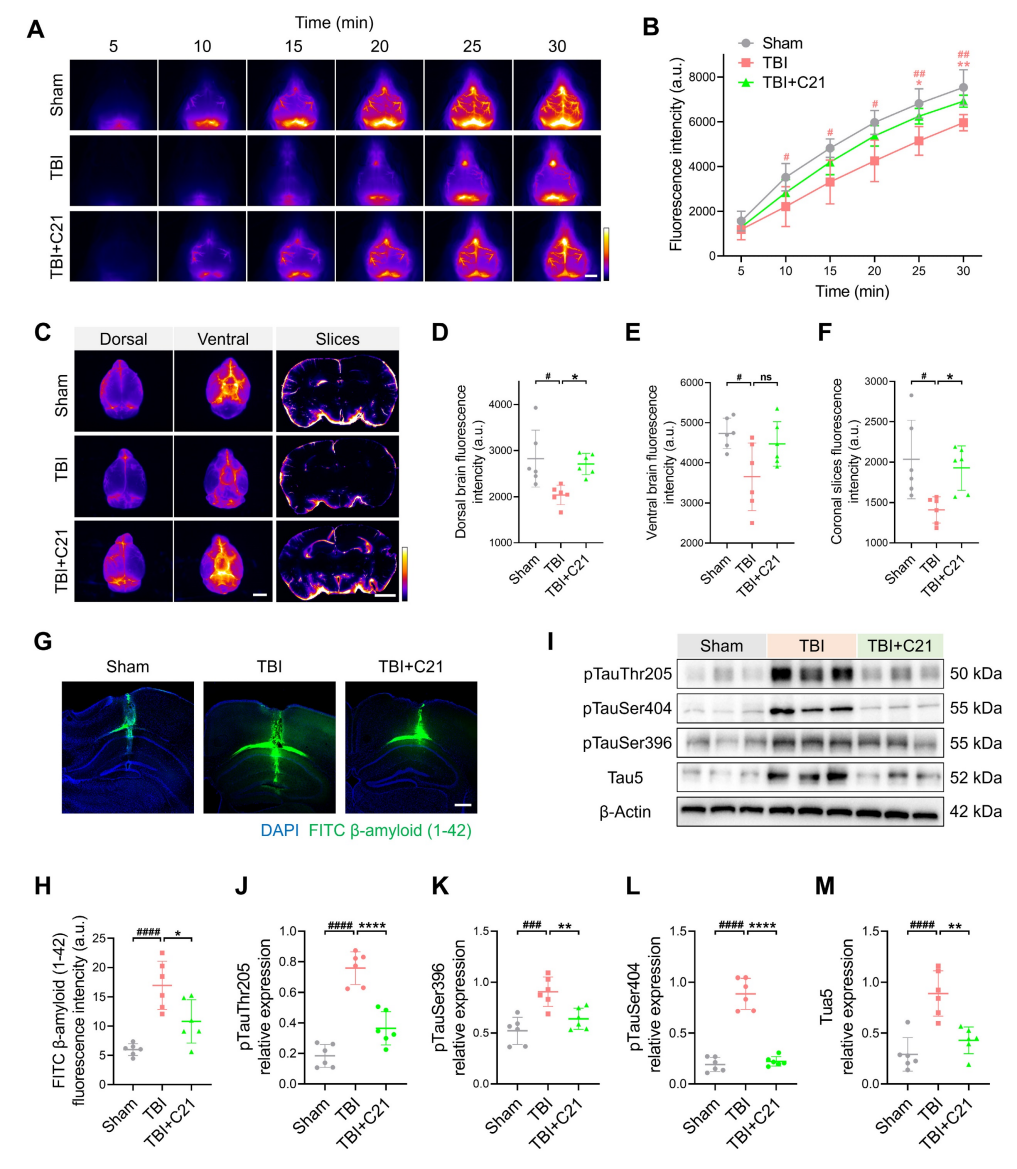
** AT2R activation suppressed β-amyloid and phosphorylated tau accumulation in chronic TBI mice. (A, B)** Representative transcranial NIR-II images (A) and quantified fluorescence intensity (B) of BSA@IR-780. Scale bar = 4 mm. **(C)** Representative images of BSA@IR-780 distribution on dorsal and ventral brain surfaces, as well as in coronal slices. Scale bars: 4 mm (surfaces), 2 mm (slices). **(D-F)** Quantified fluorescence intensity on dorsal (D) and ventral (E) surfaces, and across coronal slices (F). **(G, H)** Representative images (G) and quantification (H) of FITC-labeled β-amyloid (1-42) in brain parenchyma. Scale bar = 400 μm. **(I-M)** Western blots (I) and quantification of tau markers in perilesional cortex: pTauThr205 (J), pTauSer396 (K), pTauSer404 (L), and Tau5 (M). All data are presented as mean ± SD (n = 6 per group). Statistical significance: #p < 0.05, ##p < 0.01, ###p < 0.001, ####p < 0.0001 versus sham group; *p < 0.05, **p < 0.01, ***p < 0.001, ****p < 0.0001 versus TBI+C21 group.

TBI-induced impairment of the glymphatic system persists in the chronic phase 10. 30 days after TBI, NIR-II imaging revealed persistent impairment of glymphatic influx in the TBI group. However, AT2R activation by C21 treatment restored glymphatic influx during the chronic TBI phase (Figure 6A, B). Further corroborating these findings, *ex vivo* imaging demonstrated a similar restoration of glymphatic influx (Figure 6C-F; see Figure S11 for detailed coronal slices analysis).

The accumulation of β-amyloid and phosphorylated tau following TBI has been identified as a direct contributor to the pathogenesis of neurodegenerative diseases 61, 62. To assess β-amyloid clearance efficiency, FITC-labeled β-amyloid was injected into the cerebral cortex, and its residual levels were subsequently analyzed (Figure 6G). Quantitative analysis revealed increased β-amyloid retention in TBI mice compared to sham-operated controls. Notably, C21 treatment reduced this retention (Figure 6H). Additionally, Western blot was used to evaluate phosphorylated tau levels in the perilesional cortex (Figure 6I). C21 administration post-TBI effectively attenuated phosphorylated tau accumulation, particularly at the Thr205, Ser404, and Ser396 phosphorylation sites (Figure 6J-M).

## Discussion

**Figure 7:**
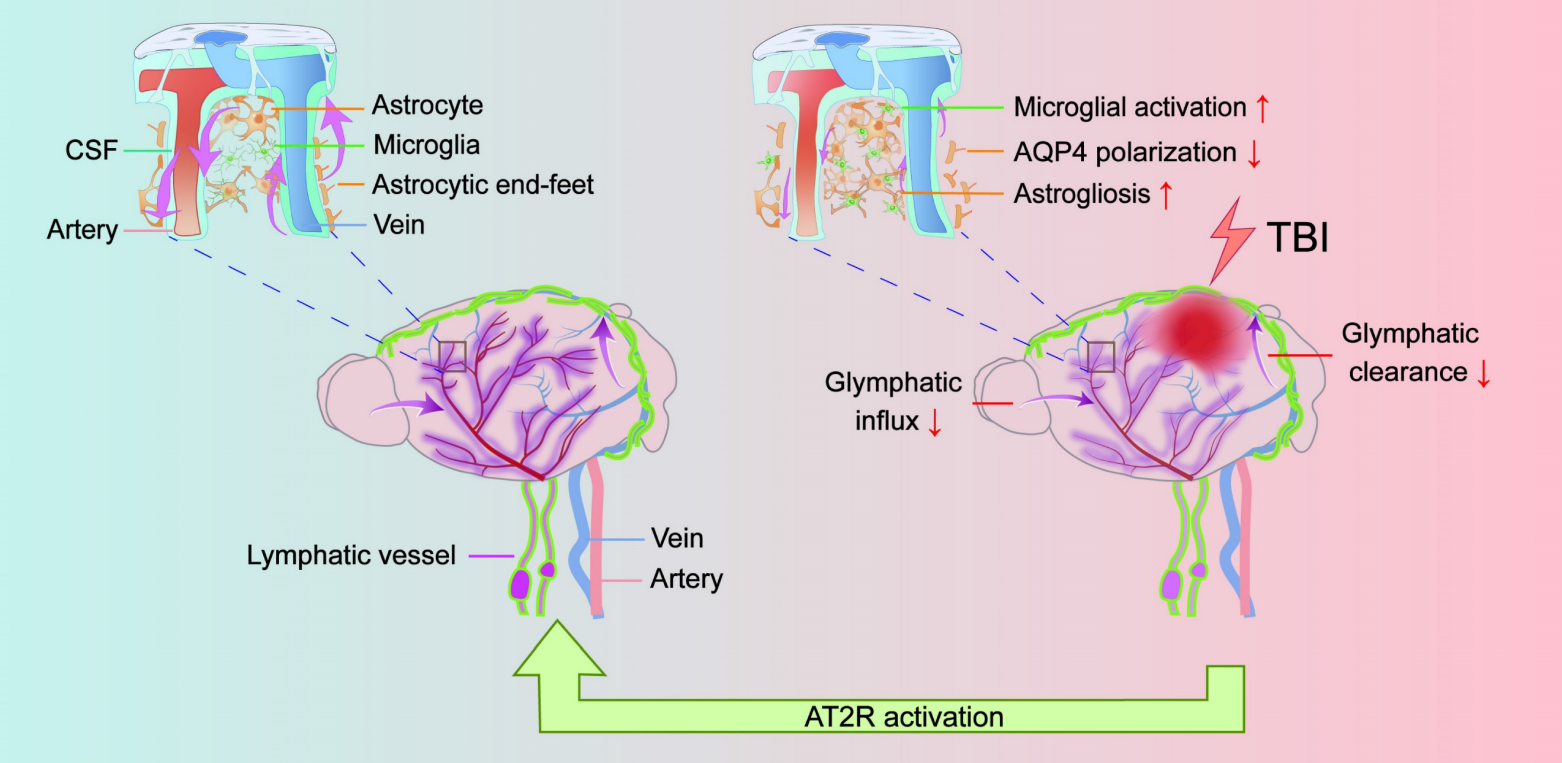
** TBI impairs glymphatic transport and is counteracted by AT2R activation.** Under physiological conditions, CSF flows from the subarachnoid space into the brain parenchyma through periarterial spaces, exchanges solutes with ISF, and ultimately drains via perivenous pathways (left panel). However, TBI induces structural damage to the glymphatic system, impairing glymphatic influx and clearance. Mechanistically, post-TBI glymphatic dysfunction arises primarily from reactive astrogliosis-mediated perivascular structural remodeling and loss of AQP4 polarization at astrocytic end-feet (right panel). Critically, C21-mediated AT2R activation reverses these pathological changes, restoring glymphatic transport post-TBI. Furthermore, AT2R activation suppresses neuroinflammatory responses, reduces neurotoxic protein accumulation, and improves neurological recovery.

This study aimed to investigate the therapeutic potential of C21 in regulating glymphatic system function following TBI. Leveraging the unique physicochemical properties of NIR-II probes, we developed a multimodal NIR-II imaging platform to visualize both glymphatic influx and glymphatic clearance, enabling real-time dynamic monitoring of glymphatic transport *in vivo*. Our data demonstrated that C21-mediated AT2R activation not only effectively restored glymphatic transport but also significantly reduced neuroinflammation, β-amyloid and phosphorylated tau accumulation, and neurological functional impairments compared with untreated TBI mice (Figure 7).

TBI impairs the function of glymphatic system through mechanical impact and neuroinflammatory pathways, manifesting as reduced AQP4 polarization and obstructed CSF-ISF circulation. These disruptions lead to diminished clearance of metabolic waste and neurotoxic proteins (e.g., β-amyloid, phosphorylated tau), ultimately contributing to neurological dysfunction 11, 21. While the RAS primarily systemically regulates cardiovascular homeostasis, its dysregulation is implicated in hypertension and cardiovascular disorders. Notably, the localized brain RAS plays a pivotal role in neurological pathologies 63, 64. In TBI, activation of the brain RAS via AT1R exacerbates injury and pathological progression. Conversely, AT2R, though minimally expressed in the adult brain under physiological conditions, demonstrates significant upregulation during ischemic, inflammatory, and neurodegenerative states, reflecting its adaptive role in injury repair. Studies have demonstrated that C21-mediated AT2R activation alleviates neuroinflammation, cerebral edema, and blood-brain barrier disruption in TBI mice, while concurrently improving myocardial fibrosis and increasing left ventricular ejection fraction 30, 31. Furthermore, C21 treatment in stroke mice suppresses neuroinflammation, decreases phosphorylation of tau at Ser202/Thr205 and β-amyloid accumulation in ipsilateral hemispheres, and enhances cognitive recovery 33. These neuroprotective mechanisms of AT2R activation provide a foundational rationale for its role in glymphatic restoration post-TBI. In this study, we employed NIR-II imaging technology to investigate AT2R-mediated regulation of glymphatic transport following TBI.

To investigate the AT2R modulation of post-TBI glymphatic influx, we first employed NIR-II imaging to track the CSF-mediated transport dynamics of BSA@IR-780 from the subarachnoid space to the brain parenchyma. The whole-brain macroscopic visualization capability of NIR-II imaging enabled precise mapping of tracer influx through the PVS across cortical surfaces. Our analyses revealed that TBI substantially compromises glymphatic influx, manifesting as a significantly delayed tracer influx. Crucially, C21-mediated AT2R activation effectively restored the influx of BSA@IR-780 and increased its flow speed in the PVS. NIR-II imaging was also employed to non-invasively monitor the drainage of BSA@IR-780 into the superficial CLNs. The results confirmed that AT2R activation enhances drainage into superficial CLNs. This dynamic macroscopic imaging approach provides clearer visualization of glymphatic transport impairment induced by TBI, while highlighting the protective effects of AT2R activation in restoring glymphatic drainage function post-TBI.

In addition, this study introduced an innovative approach using NIR-II probes to monitor the dynamic parenchymal clearance process in real-time, including albumin-escaping IR-808Ac capable of direct and real-time visualization of solute clearance in the brain parenchyma, and albumin-seeking IR-808 designed to track the dynamic of clearance solute from the brain into the blood circulation. This method provides a more precise and less invasive evaluation of glymphatic clearance in mice. Our results showed that the increased clearance efficiency of IR-808Ac in the brain parenchyma and the elevated fluorescence intensity of IR-808 in the femoral vein clearly indicated that AT2R activation enhanced glymphatic clearance following TBI. This innovative approach advances our understanding of the glymphatic clearance mechanism and highlights the therapeutic potential of AT2R activation in mitigating TBI-induced impairment of glymphatic clearance function.

Studies have established that TBI triggers diffuse reactive astrogliosis, culminating in hypertrophic glial scarring. This process disrupts perivascular AQP4 polarization and spatial distribution, representing a primary pathophysiological mechanism underlying post-TBI glymphatic system impairment 20. Previous research indicated that AT2R activation suppresses post-stroke astrogliosis and ameliorates neuroinflammatory responses in 5XFAD murine models 33. Furthermore, in angiotensin II-induced oxidative stress and inflammatory responses in astrocytes, AT2R activation attenuated reactive oxygen species overproduction, mitigated mitochondrial dysfunction, and inhibited NFκB-driven inflammatory cascades 65. Our experimental findings demonstrated that AT2R activation restored AQP4 polarization and attenuated the reactive astrogliosis following TBI. These effects provide mechanistic insight into AT2R-mediated protection against TBI-induced glymphatic dysfunction.

Blood pressure regulation is crucial following TBI, as hemodynamic instability may disrupt cerebral arterial pulsations and subsequently impair the glymphatic system function 58. Although preclinical studies indicate that C21 attenuates angiotensin II-induced hypertension 66, Timaru-Kast et al. reported that neither low nor high C21 doses affected blood pressure in TBI mice 67. Our findings demonstrated that C21 administration did not markedly alter MAP, suggesting that its protective effects may not involve blood pressure modulation. In addition, studies have demonstrated an inverse correlation between glymphatic influx and heart rate, independent of respiratory rate 59. Another study showed that CSF dynamics have been associated with respiratory rate 68. Our study revealed that C21 administration did not significantly alter the heart rate or respiratory rate in TBI mice. These findings suggest that the restoration of the glymphatic system function by C21 in TBI occurs independently of alterations in heart rate or respiratory rate regulation.

The neuroinflammatory response following TBI involves complex interactions with glymphatic dysfunction. Post-TBI inflammatory factors impair AQP4 polarization, which exacerbating glymphatic transport impairment and amplifying neuroinflammation 11. As key immune effectors in the central nervous system, microglia rapidly proliferate and display characteristic morphological activation in perilesional areas after TBI, characterized by shortened branches and reduced terminal endpoints 69. Our cytoskeletal analysis demonstrated that C21 treatment significantly inhibited microglial activation in the perilesional cortex. Furthermore, RNA-seq analysis revealed that C21 administration effectively suppressed neuroinflammatory responses in the perilesional cortex, as evidenced by the downregulation of neuroinflammatory signaling pathways. In addition, RNA-seq analysis revealed that the protective mechanism of C21 in TBI involves the ERK pathway. Specifically, TBI induces sustained activation of the ERK pathway, leading to a dramatic increase in phosphorylated ERK levels that exacerbates secondary damage. Previous studies demonstrated that AT2R activation inhibits ERK activity via SHP1 tyrosine phosphatase activation 70. Our results indicated that AT2R activation suppresses ERK1/2 phosphorylation in acute TBI mice. Crucially, ERK inhibition has been found protective in brain injury, suppressing neurotoxic astrocyte polarization, attenuating neuroinflammatory responses, reducing blood-brain barrier disruption, and promoting neurological function recovery 60, 71-73. We found that ERK inhibition facilitated the restoration of AQP4 polarization and CBF in acute TBI mice. Collectively, these results suggested that the effects of AT2R activation on restoring AQP4 polarization and CBF might be mediated through ERK inhibition.

Evidence indicates that TBI induces persistent glymphatic system impairment in murine models, with functional deficits persisting up to 6 months post-injury 10. Persistent dysfunction of glymphatic transport promotes β-amyloid and phosphorylated tau accumulation, thereby contributing to long-term neurological deficits 21. Our experimental data corroborate these findings by revealing persistent impairment of glymphatic drainage efficiency at 30 days post-TBI. Crucially, C21-mediated AT2R activation maintained glymphatic transport function during the chronic phase. The mechanistic significance of these findings is further underscored by AT2R activation reducing β-amyloid and phosphorylated tau accumulation. Behavioral assessments substantiated the relevance of this intervention, demonstrating significant amelioration of TBI-induced motor coordination deficits and cognitive dysfunction. Thus, our findings demonstrate that AT2R-targeted intervention disrupts the pathological cascade of chronic glymphatic impairment following TBI, providing sustained protection against neuropathological progression and functional deterioration.

## Conclusion

In this study, we employed NIR-II imaging to dynamically visualize glymphatic transport in mice, showing that C21-mediated AT2R activation enhanced glymphatic influx and promoted glymphatic clearance in TBI mice. Mechanistically, AT2R activation restored AQP4 polarization and simultaneously suppressed reactive astrogliosis, microglial activation, and neuroinflammatory responses. Furthermore, AT2R activation enhanced β-amyloid clearance efficiency and reduced phosphorylated tau accumulation, thereby contributing to improved post-traumatic neurological recovery. Collectively, these findings establish the critical role of AT2R in restoring post-TBI glymphatic function and identify it as a promising therapeutic target for TBI.

## Supplementary Material

Supplementary materials and methods, figures.

## Abbreviations

TBI: traumatic brain injury
CSF: cerebrospinal fluid
ISF: interstitial fluid
AQP4: aquaporin-4
PVS: perivascular spaces
GFAP: glial fibrillary acidic protein
RAS: angiotensin system
AT1R: angiotensin II type 1 receptor
AT2R: angiotensin II type 2 receptor
CBF: cerebral blood flow
C21: compound 21
NIR-II: near-infrared II
CLNs: cervical lymph nodes
ISO: isoflurane
PBS: phosphate buffered saline
mNSS: modified neurological severity score
NOR: novel object recognition
OFT: open field test
MWM: Morris water maze
CM: cisterna magna
DEX: dexmedetomidine
PFA: paraformaldehyde
MAP: mean arterial pressure
SBP: systolic blood pressure
DBP: diastolic blood pressure
RNA-seq: RNA sequencing
SD: standard deviation
DEGs: differentially expressed genes
