# Supplementary material for: Enhancing glymphatic transport through angiotensin II type 2 receptor activation promotes neurological recovery after traumatic brain injury
Source: Theranostics. 2025 Sep 3;15(18):9775–92. doi: 10.7150/thno.117743 (PMC12486419; doi:10.7150/thno.117743)
Supplement: Supplementary file 1 — Supplementary materials and methods, figures. [file thnov15p9775s1.pdf]

Supporting Information

Enhancing glymphatic transport through angiotensin II type 2 receptor activation promotes neurological recovery after traumatic brain injury

Xiaoyu Zhang[1], Bin Sun[2], Wenzhong Li[2], Tianyi Liu[3], Wenchen Li[1], Bo Chen[1], Chuan He[1], Qin Liu[3], Shoujun Zhu[2]*, Haifeng Wang[1]*

1. Department of Neurosurgery, The Second Hospital of Jilin University, Changchun, 130041, P. R. China
2. Joint Laboratory of Opto-Functional Theranostics in Medicine and Chemistry, The First Hospital of Jilin University, Changchun, 130021, P. R. China
3. Department of Neurosurgery, The First Hospital of Jilin University, Changchun, 130021, P. R. China

*Corresponding authors: sjzhu@jlu.edu.cn (S. Zhu), hfwang@jlu.edu.cn (H. Wang)

Supplementary figures

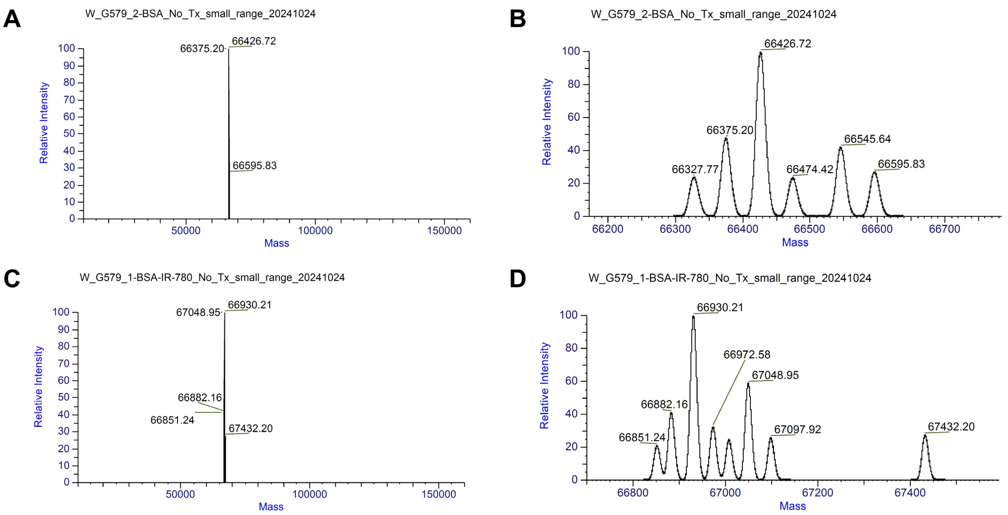

Figure S1. High-resolution mass spectrometry of the free BSA (A, B) and BSA@IR-780 (C, D).

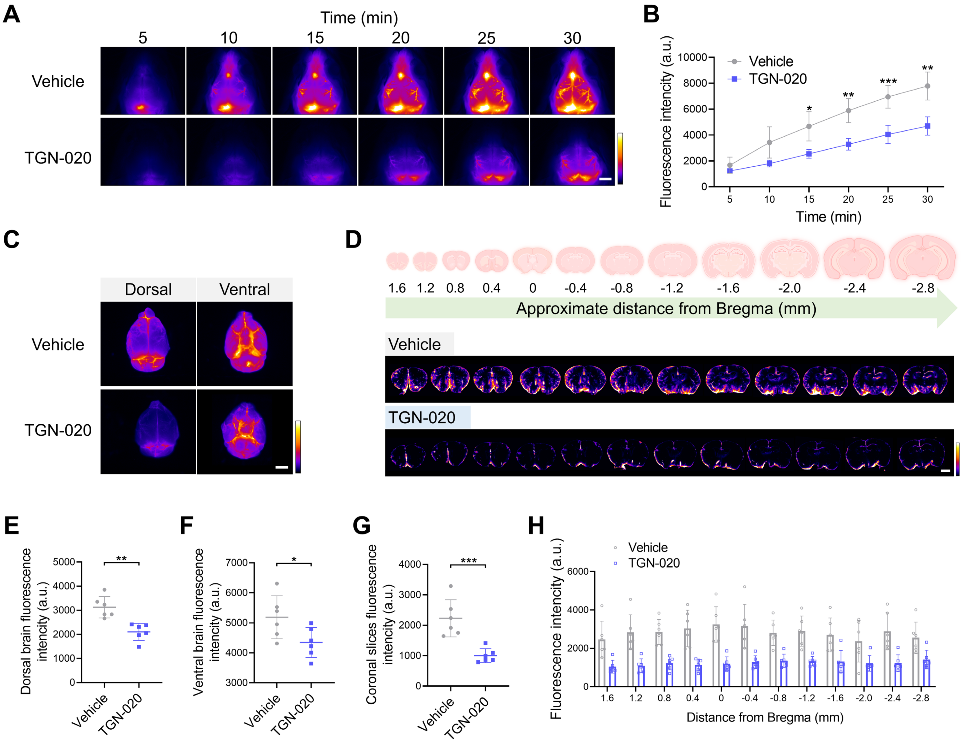

Figure S2. TGN-020 inhibited glymphatic influx in mice. **(A, B)** High-contrast transcranial imaging (A) and quantification of fluorescence intensity (B) for BSA@IR-780. Scale bar = 4 mm. **(C)** Representative images of BSA@IR-780 distribution on dorsal and ventral brain surfaces. Scale bar = 4 mm. **(D)** Representative images of

coronal slices at indicated bregma distances. Scale bar = 2 mm. **(E, F)** Quantified fluorescence intensity on the dorsal (E) and ventral (F) surfaces. **(G, H)** Quantified fluorescence intensity across all slices (G) and per slice (H). All data are presented as mean ± SD (n = 6 per group). Statistical significance: *p < 0.05, **p < 0.01, ***p < 0.001, ****p < 0.0001.

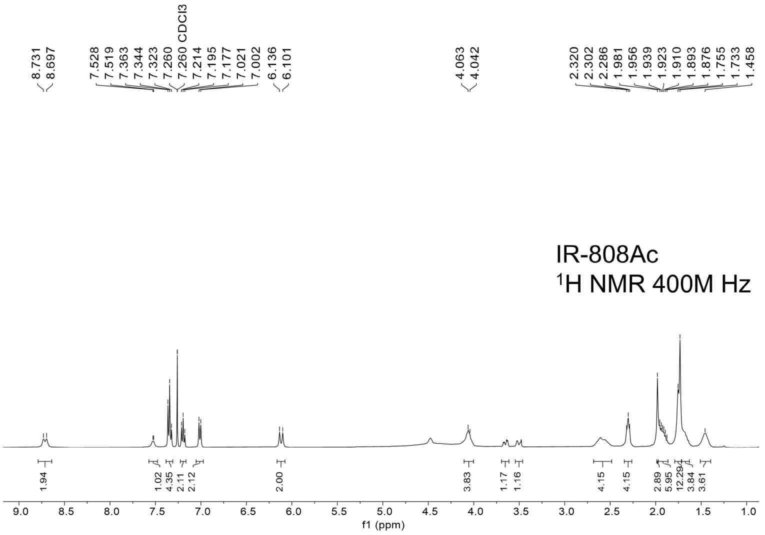

Figure S3. ^{1}H NMR Spectra of IR-808Ac [1].

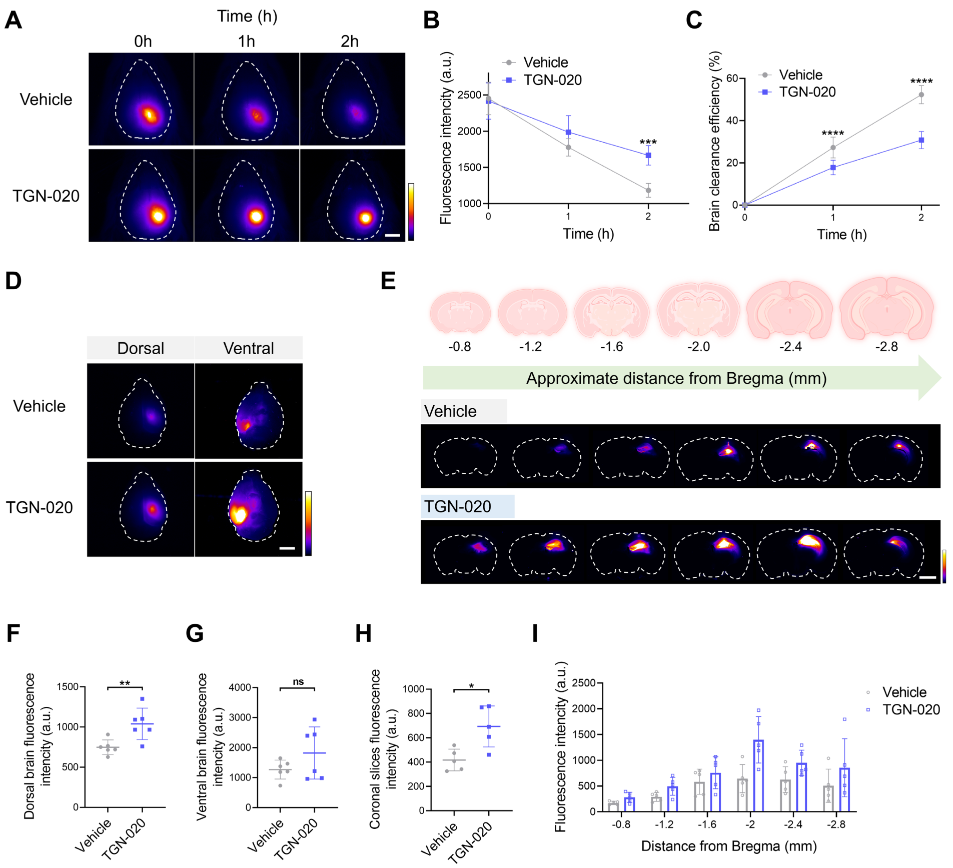

Figure S4. TGN-020 inhibited glymphatic clearance in mice. **(A-C)** High-contrast transcranial NIR-II imaging (A) and fluorescence intensity analysis of IR-808Ac (B, C). Scale bar = 4 mm. **(D)** Representative images of IR-808Ac distribution on dorsal and ventral brain surfaces. Scale bar = 4 mm. **(E)** Representative images of coronal slices at indicated bregma distances. Scale bar = 2 mm. **(F, G)** Quantified fluorescence intensity on the dorsal (F) and ventral (G) surfaces. **(H, I)** Quantified fluorescence intensity across all slices (H) and per slice (I). All data are presented as mean ± SD (n = 5 - 6 per group). Statistical significance: *p < 0.05, **p < 0.01, ***p < 0.001, ****p < 0.0001.

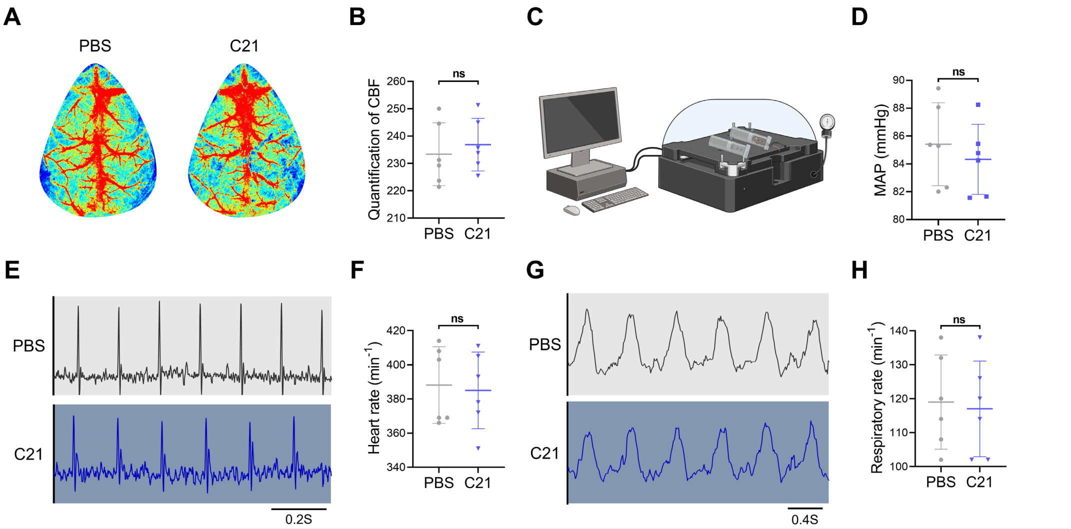

Figure S5. Monitoring of cerebral blood flow (CBF), mean arterial pressure (MAP), heart rate and respiratory rate in mice after intraperitoneal injection of C21 or PBS for 2 h. **(A)** Representative images showing the CBF for all groups. **(B)** Quantification of CBF. **(C, D)** Measurements and quantification of MAP. **(E)** Representative images of heart rate for all groups. **(F)** Quantification of heart rate. **(G)** Representative images of respiratory rate for all groups. **(H)** Quantification of respiratory rate. All data are presented as mean ± SD (n = 6 per group). Statistical significance: *p < 0.05, **p < 0.01, ***p < 0.001, ****p < 0.0001.

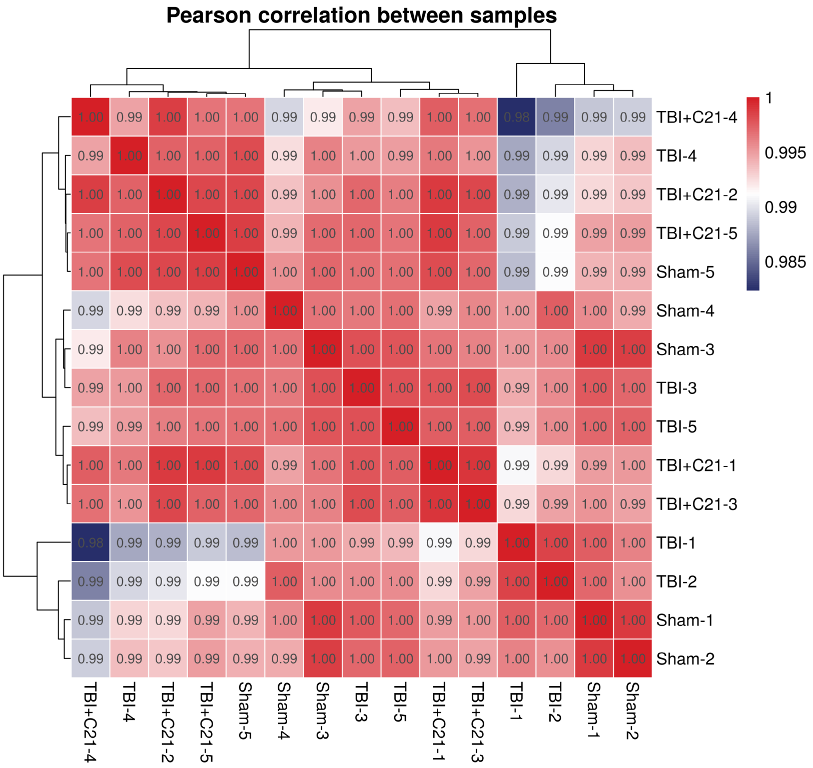

Figure S6. The assessment of the correlation between gene expression levels among samples (n = 5 per group).

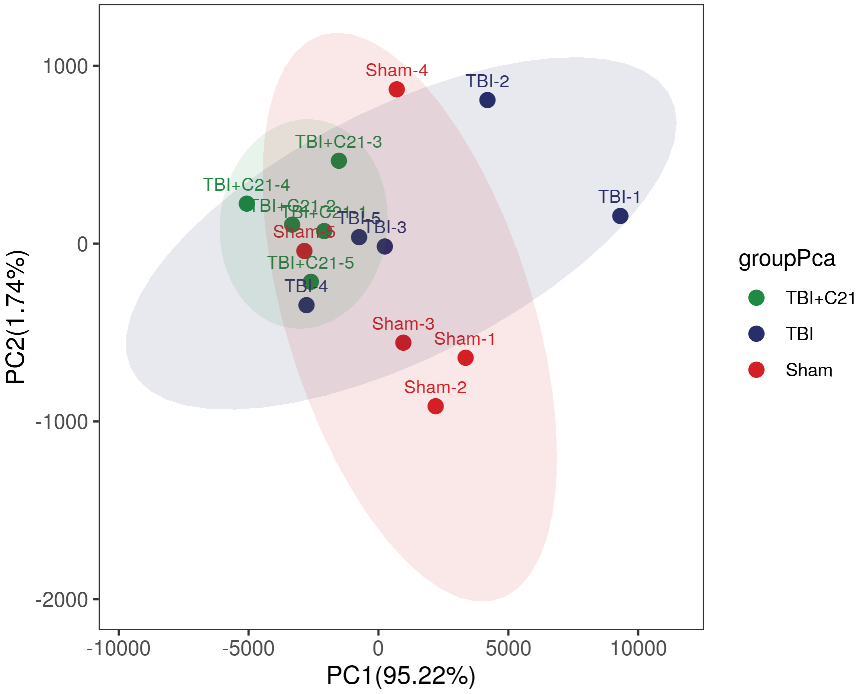

Figure S7. Principal component analysis plot (n = 5 per group).

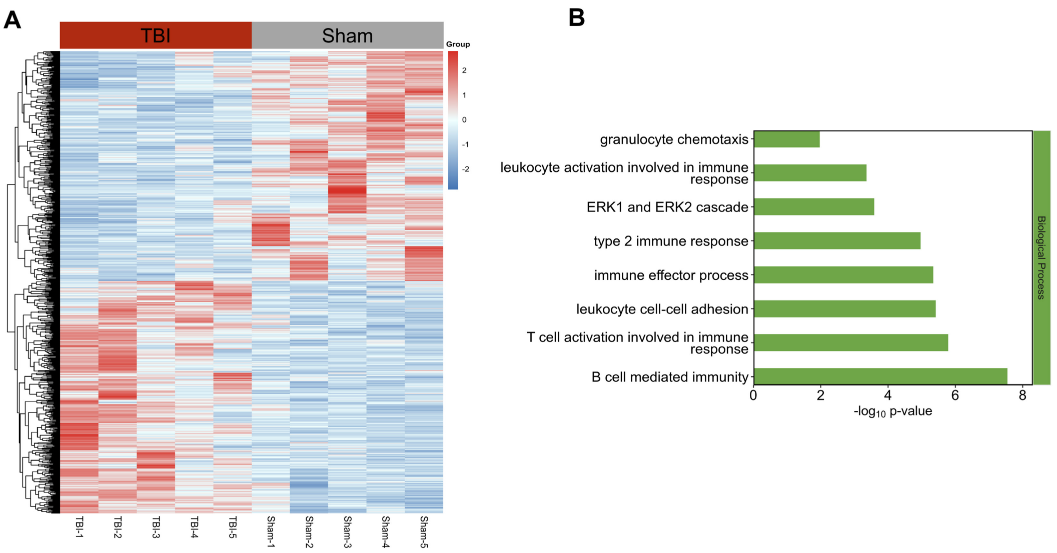

Figure S8. **(A)** Cluster map of the DEGs among TBI group and Sham group. **(B)** Up-regulated biological process of gene ontology function enrichment of the DEGs in the TBI group compared with the sham group. n = 5 per group.

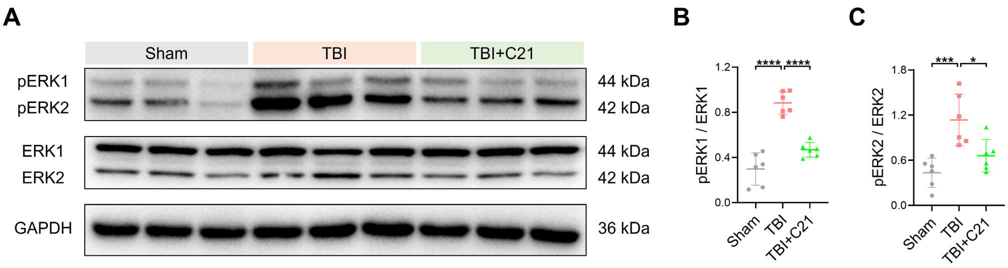

Figure S9. AT2R activation reduced the activation of ERK1/2 in acute TBI mice. **(A)** Western blot images of phospho-ERK1/2 (pERK1/2) and total ERK1/2 in the perilesional cortex. **(B, C)** Quantitative analysis of ERK1/2 phosphorylation: pERK1/ERK1 ratio (B) and pERK2/ERK2 ratio (C). All data are presented as mean ± SD (n = 6 per group). Statistical significance: *p < 0.05, **p < 0.01, ***p < 0.001, ****p < 0.0001.

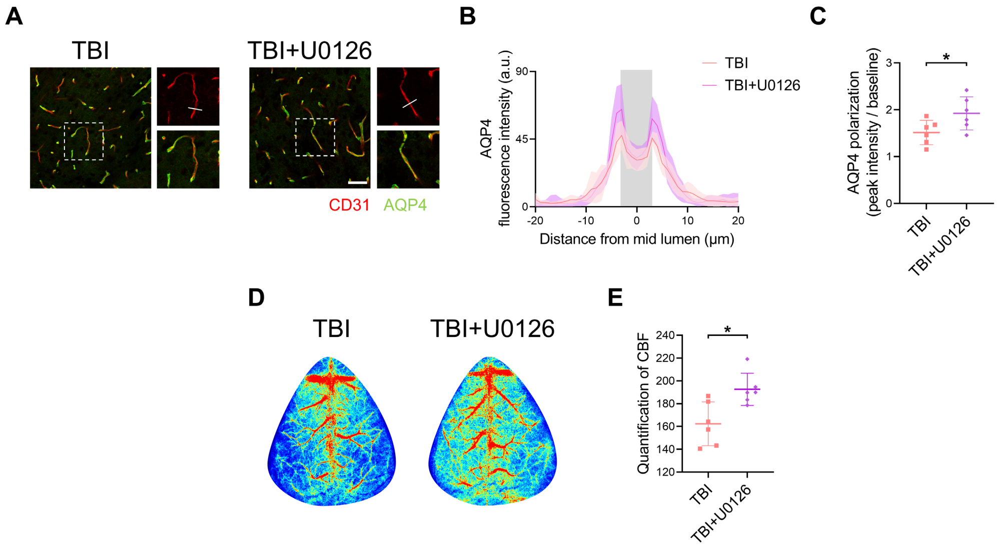

Figure S10. Inhibition of the ERK pathway restored AQP4 polarization and CBF in acute TBI mice. **(A)** Representative images of AQP4 (green) and CD31 (red) immunofluorescence staining. Scale bar = 50 μm. **(B)** Quantification of AQP4 fluorescence intensity plot along the white line in (A). **(C)** Quantification of AQP4 polarization (AQP4 polarization = vessel AQP4 peak intensity / average baseline intensity). **(D)** Representative images showing the CBF for all groups. (E) Quantification of CBF. All data are presented as mean ± SD (n = 6 per group). Statistical significance: *p < 0.05, **p < 0.01, ***p < 0.001, ****p < 0.0001.

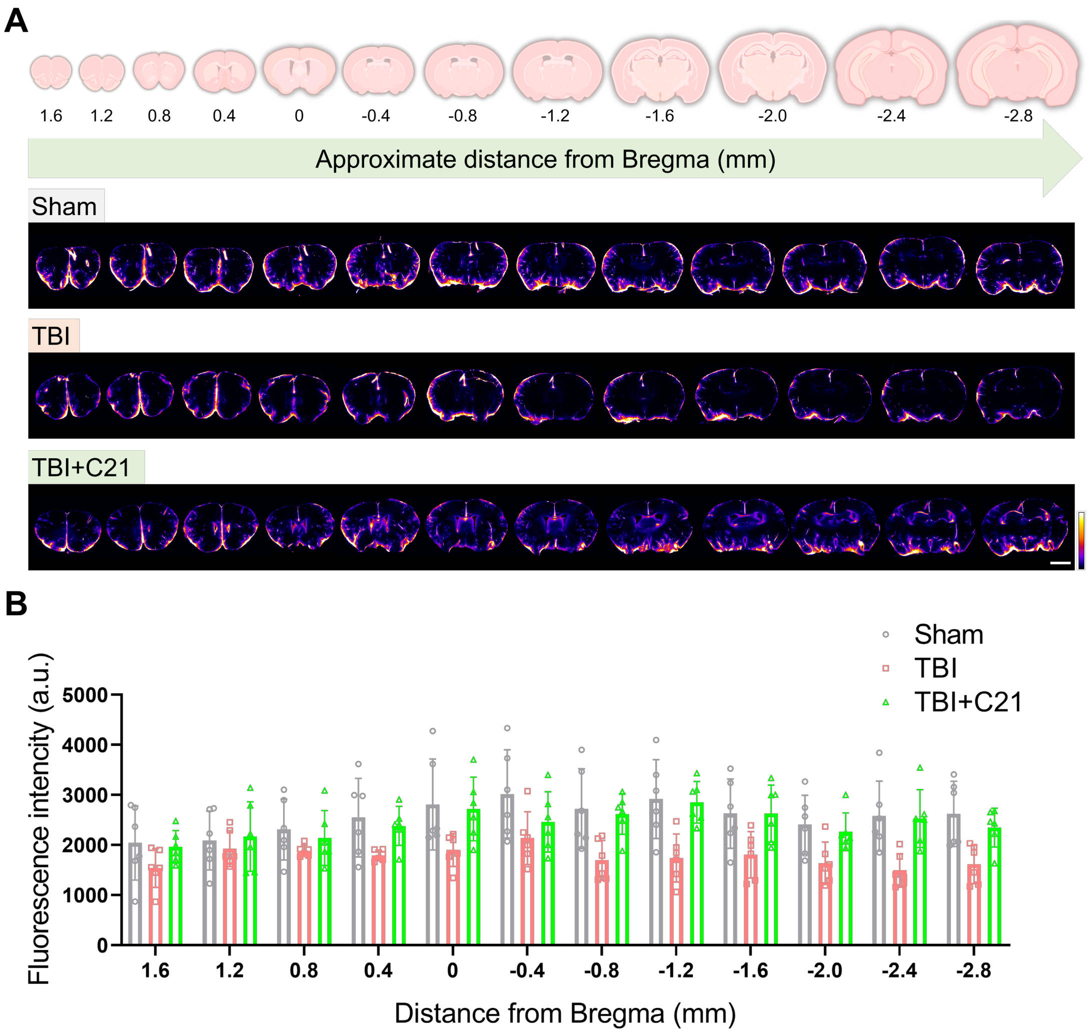

Figure S11. Evaluation of BSA@IR-780 distribution in coronal slices of chronic TBI mice. **(A)** Representative images of coronal slices at the indicated distances from bregma. Scale bar = 2 mm. **(B)** Quantified fluorescence intensity of per slice. All data are presented as mean ± SD (n = 6 per group).

Supplementary methods

Modified neurological severity score (mNSS)

Neurological deficits were quantitatively evaluated using the mNSS, a validated composite assessment tool integrating motor function, sensory response, reflex integrity, and balance capability. Systematic evaluations were conducted at 3, 5, and 7 days post-TBI to monitor temporal patterns of recovery. The mNSS employs an 18-point ordinal scale in which increasing scores correlate with the severity of neurological impairment (0 = normal function, 18 = maximal deficit). Trained investigators blinded to experimental groups performed all assessments under standardized environmental

conditions, with three consecutive trials averaged per time point.

Rotarod test

Motor coordination and dynamic balance were quantitatively evaluated using an automated rotarod system (IITC Life Science, USA) following established protocols. The assessments were performed on days 3, 5, and 7 post-TBI. Mice underwent a pre-acclimatization protocol consisting of 300-s trials at constant low-speed rotation (5–10 RPM) 24 h prior to baseline testing. During experimental sessions, the apparatus was programmed with an accelerating paradigm (5–40 RPM over 300 s), with three consecutive trials per animal conducted in a randomized order. Latency to fall was recorded and averaged per mouse.

Open field test

The test was conducted in a 40 cm × 40 cm × 40 cm arena, which was divided into central and peripheral zones. The number of times the animals crossed between zones and the time spent in each zone during the observation period were recorded to assess spontaneous locomotor activity. On the 10th day post-TBI, mice from each group were individually placed in an open-field apparatus. Each mouse was tracked via video recording for 5 min. After each trial, urine and feces were removed, and the apparatus was wiped with ethanol to eliminate odor residue. Data were analyzed using the Noldus EthoVision animal trajectory-tracking system.

Novel object recognition (NOR) test

First, each mouse was placed in a 40 cm × 40 cm × 40 cm open-field arena containing two identical cylindrical objects (A1 and A2) fixed symmetrically. The animal's activity was recorded for 5 min using a video tracking system. 6 h post-initial exposure, one cylindrical object (A2) was replaced with a novel rectangular object (B), and the mouse was re-tested under identical conditions. The Noldus EthoVision XT trajectory tracking system was utilized to quantify exploration time directed toward novel versus familiar objects, enabling assessment of recognition memory. The NOR index was calculated as:

NOR index (%) = [Time exploring novel object / (Time exploring novel object + Time exploring old object)] × 100

This method provides a statistical measure of the mouse's ability to explore new versus old objects.

Morris water maze (MWM) test

Mice were tested in a circular water maze (diameter: 120 cm; water depth: 30 cm; temperature maintained at 20–22°C) rendered opaque with non-toxic white tempera paint. During the 5-day acquisition phase, a submerged platform (5 mm below water surface) was fixed in the target quadrant. Each trial began by placing the mouse facing the wall at one of four randomized start positions, allowing 60 s to locate the platform. Mice that failed to locate the platform within 60 s were guided to it and permitted a 15-s consolidation period. Escape latency and swim paths were recorded using the Noldus EthoVision XT system. On day 6, the hidden platform was removed, and the mice were randomly placed in the pool to swim and explore for 60 s. We recorded the number of times each mouse crossed the location of the hidden platform, the time spent in the quadrant where the platform was previously located, and their swim trajectories using the Noldus tracking system.

Drug administration

TGN-020 (HY-W008574, MedChemExpress), a specific AQP4 inhibitor used for pharmacological glymphatic system blockade, was dissolved in 20% sulphobutylether-β-cyclodextrin (C871854, Macklin) in water for injection, as previously described [2, 3]. TGN-020 (250 mg/kg) was administered intraperitoneally 15 min before NIR-II imaging.

U0126 (HY-12031A, MedChemExpress), an ERK1/2 inhibitor [4-6], was prepared in 5% DMSO in saline and administered intraperitoneally to mice 1 h after TBI at a dose of 30 mg/kg, followed by two additional doses at 24 and 48 h post-injury. Control mice received an equivalent volume of the DMSO/saline vehicle.

Quantification of microglia activation

The quantitative method used to analyze microglial activation was adapted from previous studies [7, 8]. Images were captured using a fluorescence microscope with a 40x objective lens and analyzed using ImageJ with the AnalyzeSkeleton (2D/3D) plugin. First, an unsharp masking method was applied to the image (pixel radius = 3, masking weight = 0.6) to eliminate pepper noise caused by insufficient masking sharpness. The image was then converted to binary using a threshold function. To enhance the microglial outline, the image was closed, and the speckle noise removal. Subsequently, the image was skeletonized and analyzed using the skeleton analysis function. Microglial activation was assessed by measuring the average branch length and endpoint voxels for each cell type.

References

1. Du Y, Xu J, Zheng X, Dang Z, Zhu N, Jiang Z, et al. NIR-II Protein-Escaping Dyes Enable High-Contrast and Long-Term Prognosis Evaluation of Flap Transplantation. Adv Mater. 2024; 36: e2311515.
2. Harrison IF, Ismail O, Machhada A, Colgan N, Ohene Y, Nahavandi P, et al. Impaired glymphatic function and clearance of tau in an Alzheimer's disease model. Brain. 2020; 143: 2576-93.
3. Kress BT, Iliff JJ, Xia M, Wang M, Wei HS, Zeppenfeld D, et al. Impairment of paravascular clearance pathways in the aging brain. Ann Neurol. 2014; 76: 845-61.
4. Maddahi A, Edvinsson L. Cerebral ischemia induces microvascular pro-inflammatory cytokine expression via the MEK/ERK pathway. J Neuroinflammation. 2010; 7: 14.
5. Wang F, Xia Z, Sheng P, Ren Y, Liu J, Ding L, et al. Targeting the Erk1/2 and autophagy signaling easily improved the neurobalst differentiation and cognitive function after young transient forebrain ischemia compared to old gerbils. Cell Death Discov. 2022; 8: 87.
6. Maddahi A, Edvinsson L. Enhanced expressions of microvascular smooth muscle receptors after focal cerebral ischemia occur via the MAPK MEK/ERK pathway. BMC Neurosci. 2008; 9: 85.

7. Hsu SJ, Zhang C, Jeong J, Lee SI, McConnell M, Utsumi T, et al. Enhanced Meningeal Lymphatic Drainage Ameliorates Neuroinflammation and Hepatic Encephalopathy in Cirrhotic Rats. Gastroenterology. 2021; 160: 1315-29.e13.

8. Rodrigo R, Cauli O, Gomez-Pinedo U, Agusti A, Hernandez-Rabaza V, Garcia-Verdugo JM, et al. Hyperammonemia induces neuroinflammation that contributes to cognitive impairment in rats with hepatic encephalopathy. Gastroenterology. 2010; 139: 675-84.
